# ClinicalGAN: powering patient monitoring in clinical trials with patient digital twins

**DOI:** 10.1038/s41598-024-62567-1

**Published:** 2024-05-28

**Authors:** Shantanu Chandra, P. K. S. Prakash, Subhrajit Samanta, Srinivas Chilukuri

**Affiliations:** 1 ZS, 2nd Floor, MFAR Manyta Tech park, Phase IV, Manayata Tech Park, Nagavara, Bengaluru, Karnataka India; 2 ZS, 1560 Sherman Ave, Evanston, IL 60201 USA

**Keywords:** Clinical trials, Monitoring, Generative AI, GAN, Computer science, Information technology

## Abstract

Conducting clinical trials is becoming increasingly challenging lately due to spiraling costs, increased time to market, and high failure rates. Patient recruitment and retention is one of the key challenges that impact 90% of the trials directly. While a lot of attention has been given to optimizing patient recruitment, limited progress has been made towards developing comprehensive clinical trial monitoring systems to determine patients at risk and potentially improve patient retention through the right intervention at the right time. Earlier research in patient retention primarily focused on using deterministic frameworks to model the inherently stochastic patient journey process. Existing generative approaches to model temporal data such as TimeGAN or CRBM , face challenges and fail to address key requirements such as personalized generation, variable patient journey, and multi-variate time-series needed to model patient digital twin. In response to these challenges, current research proposes ClinicalGAN to enable patient level generation, effectively creating a patient’s digital twin. ClinicalGAN provides capabilities for: (a) patient-level personalized generation by utilizing patient meta-data for conditional generation; (b) dynamic termination prediction to enable pro-active patient monitoring for improved patient retention; (c) multi-variate time-series training to incorporate relationship and dependencies among different tests measures captured during patient journey. The proposed solution is validated on two Alzheimer’s clinical trial datasets and the results are benchmarked across multiple dimensions of generation quality. Empirical results demonstrate that the proposed ClinicalGAN outperforms the SOTA approach by 3–4$\times$ on average across all the generation quality metrics. Furthermore, the proposed architecture is shown to outperform predictive methods at the task of drop-off prediction significantly (5–10% MAPE scores).

## Introduction

Clinical trials are an essential step towards bringing new drugs to the market and driving value for society. However, conducting clinical trials is getting increasingly challenging lately due to spiraling costs, increased time-to-market, and poor patient access. While the cost of conducting trials has increased by $4\times$ in the last two decades, Eroom’s Law indicates that the number of new drugs gaining regulatory approval per billion USD spent has reduced to half in the last decade. Patient recruitment and retention are the key challenges that impact 90% of the trials, directly affecting the time-to-market and success rates of these trials. While a lot of attention has been given to improving patient recruitment through standardized frameworks^1^ and AI-based technologies^2^, not much progress has been made on patient monitoring and retention. Currently, clinical trial monitoring is largely limited to risk-based monitoring systems^3^ that focus on improving participant protections while improving data integrity. Some recent works have focused on developing predictive methods that make single point-predictions for patient attrition^4^. These methods make deterministic assumptions about the patient’s behavior during a trial,i.e., set of input variables will always point to the same output scenario. However, patient journeys are inherently stochastic in nature as trajectories are affected by (a) inherent variability in the observed variables or, (b) the presence of unobserved factors such as patient commodities, environment and therapy. Thus, two patients with the same diagnosis may exhibit different symptoms, progress at different rates, and respond differently to the same therapy in the real world. While deterministic methods are limited to capturing just the direct effect of observed variables in the system in the form of “most likely outcome” only, stochastic methods are capable of *reproducing the broader range of possibilities* observed due to the *variability in the effects of these observed or unobserved variables*. Thus, stochastic frameworks produce more accurate future states of patients under a probabilistic setting via simulations. Furthermore, predictive methods also inherit multiple limitations by design such as: (1) tend to overfit in small dataset scenarios; (2) explainability is limited by feature engineering; and (3) point predictions lead to decision boundaries being optimized for the most probable scenarios only (mode of the distribution), instead of capturing the entire breadth of distribution of observed patient journeys.

**Figure 1:**
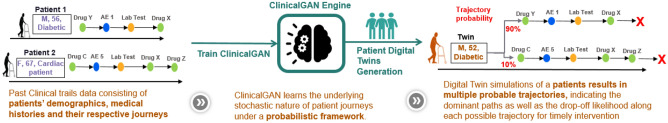
Overview of the ClinicalGAN framework to generate Patient Digital Twins (PDTs). The ClinicalGAN framework learns to simulate PDT journeys by leveraging past clinical trial data via a conditional generator. PDT simulations are stochastic in nature and thus simulate multiple possible trajectories that can be undertaken by patients based on their demographics and medical history.

Thus, to mitigate these shortcomings of existing methods, this work proposes ClinicalGAN, a patient digital twin simulation engine to model patient journeys under a probabilistic setting. ClinicalGAN is a generative architecture that provides a personalized patient-level comprehensive view of all their likely trajectories as summarized in Fig. 1. Our proposed framework first models patient journey distributions from historical data to not only learn the dominant paths, but also the other probable paths a patient can undertake at any given point while being aware of the outcomes and drop-off likelihoods along each trajectory. We then leverage this learned joint distribution to simulate the journeys of patients in current trials to understand their deviation statistics from their expected dominant paths (as inferred from historical data), and to predict their drop-off likelihood in future visits. This fundamental paradigm shift from predictive to generative methods for patient monitoring provides a more accurate, reliable and complete solution to understanding and modeling patient behavior.

Although adopting generative methods that enable stochastic simulations is the right step forward towards effective patient monitoring, it is not straightforward to model patient journeys in such a probabilistic setting. For any framework to model patient behavior in real-world data, it needs to possess three critical characteristics. Firstly, the system should be capable of personalized generation, i.e., it should be able to control the journey generation based on patient demographics, medical history as well as the trajectory so far to simulate accurate future states of the patients. Secondly, the framework should be able to handle multivariate generation. The system should be adept in modeling the spatio-temporal correlations (i.e., interaction with each other, as well as their joint evolution over time) of multiple variables typically of mixed data types (categorical, continuous, ordinal). Thirdly, the generative framework should be able to monitor termination conditions in a journey. Different trajectory simulations determine if/when the patient is likely to drop-off, and the system should be aware of the likelihood of these events for a realistic representation of real-world behavior.

However, none of the current state-of-the-art (SOTA) generative models, such as TimeGAN^5^, C-RNN-GAN^6^ and RCGAN^7^, handle all these criterion simultaneously that are critical for a patient digital twin. For instance, TimeGAN^5^, focuses mainly on the multi-variate generation aspect of the problem and fails to address the other two aforementioned dimensions. Thus, to address the above limitations of personalized monitoring as well as current SOTA generative methods, we propose ClinicalGAN, a Patient Digital Twin (PDT)-based, generative AI framework that can be used to generate patient journeys to proactively monitor their progression and predict drop-off. PDTs are virtual replicas of physical patients that capture and simulate patient’s clinical journeys and can be used to proactively monitor patients during the course of the trial. The proposed model, ClinicalGAN, is based on a class of generative models called Generative Adversarial Networks (GANs)^8^ that use adversarial training to generate realistic data. We deploy multiple learning strategies (both supervised and unsupervised) in tandem to generate high-fidelity synthetic clinical trial data using ClinicalGAN. Although recent works like RC-GAN^7^ and C-RNN-GAN^6^ also enable sampling from a learned distribution to simulate stochastic patient journeys, TimeGAN comes the closest to satisfying the required capabilities and is the current SOTA for the task by outperforming the above methods. Thus, ClinicalGAN extends TimeGAN by including:

1. Conditional generation capability to align journey simulations with patient meta-data enabling personalized generation
2. Dynamic termination prediction to enable pro-active patient monitoring for patient retention
3. Multi-objective training to support multiple real-world scenarios of journey forecasting

Additionally, in order to achieve the aforementioned tasks, this work also introduces two deep-learning novelties in the ClinicalGAN architecture that help advance the conditional sequential data generation paradigm in general:

1. Dynamic termination network—we demonstrate how a termination network can be designed to work in conjugation with a conditional generator to learn the drop-off joint distribution in relation to the evolving patient trajectory. Stabilizing the learning dynamics of this network along with other components in this architecture is the key to accomplishing the task of conditional patient-level data generation.
2. Auxiliary classifier for spatio-temporal architectures—this work introduces a novel auxiliary classifier setup for sequential auto-regressive architectures to demonstrate how this technique can be adopted from the computer vision literature, and be leveraged to improve the generation quality of patient-level medical data under a conditional generator setup.

The proposed architecture is validated on two Alzheimer’s clinical trial datasets ADNI^9^ and CODR-AD^10,11^ and the results are benchmarked across multiple dimensions of generation quality (fidelity, diversity, utility and simulation accuracy). Empirical results demonstrate that the proposed ClinicalGAN outperforms the SOTA approach by 3–4$\times$ on average across all the metrics. Additionally, experiments to test the look-ahead horizon performance of ClinicalGAN also demonstrate that it produces precise and coherent patient journeys while adhering to patients’ meta-data with consistency over distant future steps attesting to its long-term trend capture capability. Finally, the simulations via the proposed architecture outperform predictive methods by 5–10% MAPE scores at the task of patient drop-off prediction.

## Related work

ClinicalGAN is a generative model for discrete-time, multivariate, mixed-type sequence generation. This framework embodies the intersection of multiple themes of research such as probabilistic modeling in adversarial setting (GANs^8^) for sequence generation^6,7,12^ as well as sequential representation learning^13–15^.

**Univariate time series, continuous data type only** Earliest works have used variants of Fourier transforms, ARIMA/SMA, dynamic time warping (DTW) or first and second-order Markov chain models^16–20^ for the similar task of time-series generation. However, these methods can not handle multi-variate mixed data types, lack the capability of conditional modeling for personalized generation and do not perform well on panel data.

**Temporal error propagation in auto-regressive models** To address the above mentioned shortcomings of univariate models, subsequent works adopted auto-regressive deep learning methods like recurrent neural networks (RNNs) to capture the complexities of the task better. These models were capable of modeling multi-variate temporal dependencies much better. However, auto-regressive RNNs are usually trained via the maximum likelihood (MLE) procedure^21^ and are thus prone to predictive error accumulation over long sequences due to discrepancy between closed-loop training (i.e., conditioned on previous step ground truths) and open-loop inference (i.e., conditioned on their own previous step generation).

**Probabilistic deep generative models** Subsequently, the first set of generative models that gave acceptable performance at the task of modeling step-wise transition dynamics came from advanced deep learning methods summarized in Table 1. While teacher forcing^21^ and professor forcing^22^ methods addressed the shortcomings of auto-regressive methods before them, they still did not support sampling from a learned distribution. Thus, the only source of variability in output (mimicking stochastic nature to some extent) could be derived only from the output probability model.

**Table 1:** Related work summary elucidates that while different architectures incorporate different aspects of the desired characteristics, none address *all* of them together.

	Teacher-forcing^21^	Professor-forcing^22^	C-RNN-GAN^6^	RC-GAN^7^	TimeGAN^5^	ClinicalGAN (ours)
Stochastic			$\checkmark$	$\checkmark$	$\checkmark$	$\checkmark$
Multivariate mixed-data types					$\checkmark$	$\checkmark$
Latent space modeling					$\checkmark$	$\checkmark$
Sequence termination						$\checkmark$
Conditional generation (static)				$\checkmark$		$\checkmark$
Open loop (training + inference)	$\checkmark$	$\checkmark$	$\checkmark$		$\checkmark$	$\checkmark$
Close loop (training + inference)		$\checkmark$				$\checkmark$

Stochastic—model the sequential data under a probabilistic framework. Multivariate mixed data types—model spatio-temporal correlations of multiple variables of any data type (ordinal, categorical, numeric). Latent space modeling—learn the underlying data patterns in a dense latent space. Conditional generation (static)—condition the sequence generation on patient’s conditional vectors for controlled personalized generation. Sequence Termination—learn the termination triggers based on journey progression. Open loop (training/inference)—conditioned on its own previous generation at each step. Closed loop (training/inference)—conditioned on ground truth sequence at each step.

**Adversarial deep generative models** As a result, multiple subsequent studies such as RC-GAN^7^ and C-RNN-GAN^6^ inherited the GAN^8^ framework to introduce probabilistic learning paradigm for temporal data using adversarial learning. C-RNN-GAN adopts the open-loop training and inference strategy, but models only continuous data and is not equipped to handle conditional generation. RC-GAN advances this work by incorporating the conditional generation capability. However, it uses just a random seed with static conditional variables at each temporal input to produce the synthetic output at each step. Thus, it relies solely on the internal state components of the RNN to model the sequential consistency instead of training in an open/closed loop setting. This hampers its spatio-temporal modeling performance. Additionally, both these methods rely solely on unsupervised adversarial learning which the current SOTA method, TimeGAN^5^, empirically demonstrated to not be sufficient to guarantee effective modeling of the underlying data.

**Multi-objective adversarial deep generative models** TimeGAN outperformed all the above approaches by introducing multi-objective training and not relying on adversarial loss alone. It did so by employing an auto-encoder to learn the temporal dynamics in a latent space alongside adopting supervised as well as unsupervised training of the network components. However, even TimeGAN does not support conditional generation capability in its experiments (i.e., generating coherent journeys pertaining to specific individuals) as summarized in Table 1, and also suffers adversely with mode-collapse (the phenomenon of generating similar sequences repeatedly and failing to generate diverse realistic samples). Furthermore, TimeGAN is not equipped to model the varying sequence lengths of panel data, and thus resorts to generating equal length sequences only. This vastly limits its application in real-world clinical trial scenarios where patient journeys evolve stochastically over time and certain triggers (such as adverse events, etc.) can accelerate early termination.

Our proposed method tackles these existing shortcomings by modeling the termination conditions jointly with the patient meta-data (demographics and medical history) as well as journey progression to learn the likelihood of termination dynamically. Additionally, to enable personalized generation via conditional variables and stabilize GAN training, the proposed framework introduces a diversity-enhancing adversarial objective. Finally, unlike previous works, we design the ClinicalGAN architecture to be able to support multiple generation scenarios encountered in real-world use cases in a single framework (i.e., pure generative mode and journey forecasting from partial sequences mode; discussed in detail in “Conditional generator and discriminator (G and D)”). ClinicalGAN is thus equipped to be run in both open—as well as close-loop modes, both during training as well as inference phases. It is also worth noting that although the proposed method bears some resemblance to certain GAN-based approaches for semi-supervised learning^23–25^, these methods are designed for supervised classification via generation of additional unlabeled examples for training. Meanwhile, ClinicalGAN focuses on the unsupervised task of termination-aware multi-variate conditional synthetic data generation.

## Methodology

The current section discusses the design of each constituent component of the proposed ClinicalGAN architecture and their respective learning objectives.

### Problem formulation

Clinical trial datasets capture cross-sectional/static information that do not change over time (e.g., demographics, medical history, etc of patients), as well as sequential/temporal information of the patients that are time-dependent and change over the course of the trial (e.g., vitals, lab values, etc). Let $\mathcal {X}_s$ represent the vector space of these static features and $\mathcal {X}_t$ of the temporal features of patients. Let $\textbf{X}_s \in \mathcal {X}_s, \textbf{X}_t \in \mathcal {X}_t$ be random vectors that represent all the possible values that these variables can take in the real-world. These random vectors can be instantiated with specific values denoted by vectors $\textbf{x}_s$ and $\textbf{x}_t$ corresponding to information of specific patients in the population. Note that $\textbf{x}_s$ and $\textbf{x}_t$ are vectors consisting of variables of mixed data types (continuous, categorical and ordinal). For instance, $\textbf{x}_s = [54, Male, birth\_NY, diabetic\_1, smoker\_0]$ is the vector representation of static information of a patient who is a 54 year old Male born in New York who is diabetic and a non-smoker. Here, $\textbf{x}_s$ consists of one continuous variable (age), three categorical variables (gender, city of birth and diabetic indicator), and one ordinal variable (smoking level: 0 = non-smoker, 1 = occassional, 2 = regular). We can also define $\textbf{x}_t$ in a similar fashion that will represent the vitals, lab results, AEs, etc of the patient at time-step *t* in their clinical trial journey.

To extend this over a population of patients, let the individual samples of training data be indexed by $n \in {1,..., N}$, so we can denote the clinical trial training dataset as $\mathbf {\mathcal {D}} = {(\textbf{X}_{n,s}, \textbf{X}_{n,1:T_n}})$. Going forward, subscripts *n* are absorbed in the notation and omitted unless explicitly required. Our goal is to use training data $\mathcal {D}$ to learn a joint density $\hat{p}({\hat{\textbf{X}}}_s, {\hat{\textbf{X}}}_{1:T})$ that best approximates the original joint density $p({\textbf {X}}_s, {\textbf {X}}_{1:T})$ that represents how do the static and temporal variables ($\textbf{x}_s$, $\textbf{x}_{1:T}$) interact with each other to dictate the progression of patient journeys. To do so, we make use of the autoregressive decomposition as $p({\textbf {X}}_s, {\textbf {X}}_{1:T}) = p({\textbf {X}}_s) \prod _t p({\textbf {X}}_t | {\textbf {X}}_s, {\textbf {X}}_{1:t-1})$ which represents the fact that the target joint distribution of patient journeys in a clinical trial can be learned by modeling the effect of patient demographics ($\textbf{X}_s$) and their journey so far (summarized by $\textbf{X}_{t-1}$) on determining the next step in the journey ($\textbf{X}_t$) iteratively. For the task of conditional generation, the static data (${\textbf {X}}_s$) is always assumed to be given, meaning, $p({\textbf {X}}_s)$ is known. This reduces the learning objective to approximating the real $p({\textbf {X}}_t | {\textbf {X}}_s, {\textbf {X}}_{1:t-1})$ by the learned $\hat{p}({\hat{\textbf{X}}}_t | {\hat{\textbf{X}}}_s, {\hat{\textbf{X}}}_{1:t-1})$ for all time-steps *t*. Under a GAN framework, this approximation objective takes the form of the Jensen-Shannon divergence between the real density (*p*) and learned density ($\hat{p}$) yielding the objective:

1 $$\begin{aligned}min_{\hat{p}} JSD \big ( p(\textbf{X}_t | \textbf{X}_s, \textbf{X}_{1:t-1}) || \hat{p}({\hat{\textbf{X}}}_t | {\hat{\textbf{X}}}_s, {\hat{\textbf{X}}}_{1:t-1}) \big ) \end{aligned}$$

However, clinical trial data can be extremely sparse due to high cardinality and have very few data points to learn from. Such high-dimensional sparse data in a low-resource setting hinders learning due to its high degree of freedom. Thus, we transfer the entire learning procedure into a lower-dimensional manifold to aid the model to handle data sparsity. We achieve this via an auto-encoder. Auto-encoders help with feature compression while preserving the underlying relationship between the variables. Subsequently, this switch in formulation from original feature space to latent space is achieved by a minor change in the aforementioned learning objective, such that ${\textbf {X}}_s$, ${\textbf {X}}_{1:T}$ are replaced with their hidden representations ${\textbf {h}}_s \in \mathcal {H}_s$, ${\textbf {h}}_{1:T} \in \mathcal {H}_t$ respectively, where $\mathcal {H}_s$ and $\mathcal {H}_t$ represent the corresponding latent vector spaces. This yields the final learning objective to be:

2 $$\begin{aligned} min_{\hat{p}} JSD \big ( p(\textbf{H}_t | \textbf{H}_s, \textbf{H}_{1:t-1}) || \hat{p}({\hat{\textbf{H}}}_t | \textbf{H}_s, {\hat{\textbf{H}}}_{1:t-1}) \big ) \end{aligned}$$

Important assumption of this work: cause and effect may not occur in immediate succession within the patient’s journey during a clinical trial as it could take time for symptoms to manifest after the causal events. Therefore, time-ordering does not inherently imply causality. Both temporal sequences along with domain-specific knowledge is required to determine the true causal relationships within the data. As part of this research, the focus is on learning and generating patient journeys using real-world sequences of patient’s journey as obtained from clinical trial, considering both demographic and temporal relationships. We keep the temporal ordering as an important part of the framework design since during a real-world patient monitoring scenario, we do expect casual inaccuracies within the sequence with respect to the natural temporal ordering of events while predicting patient drop-off. Thus, time-order is preserved to maintain the statistical property for the generation followed by the prediction task. However, it does impact the casual interpretation at event level and is area of future research.

### Proposed architecture: Conditional ClinicalGAN

**Figure 2:**
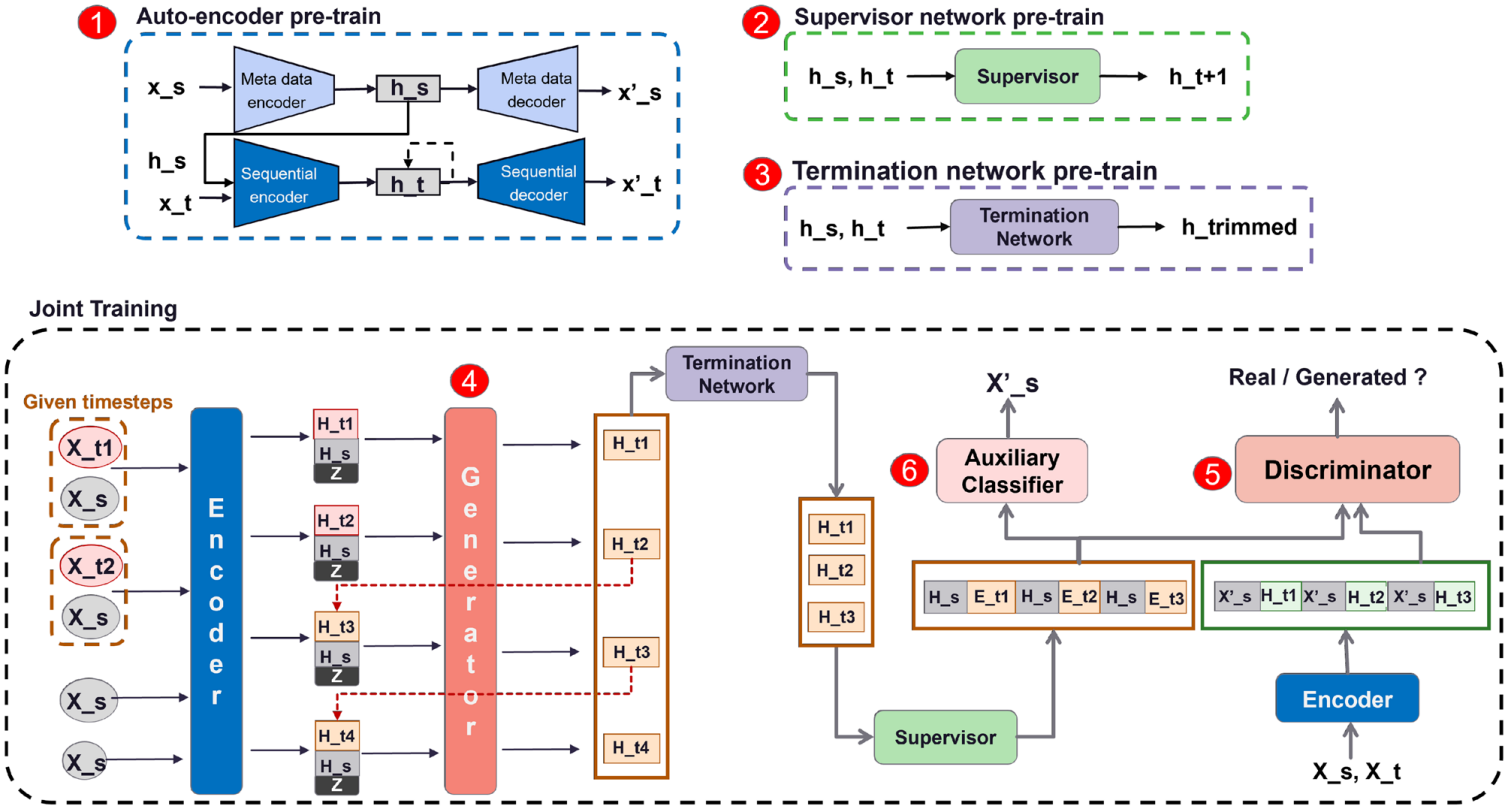
ClinicalGAN consists of six network components. The *auto-encoder (1) (AE)* transforms the data from feature space to compressed latent space; (2) *supervisor network (S)* to assist the generator with a supervised objective to further guide the learning process ; (3) *termination network* ($$T_r)$$ models the drop-off likelihood of patients over their simulated journeys; (4) *conditional generator (G)* auto-regressively generates personalized patient journeys; (5) *conditional discriminator (D)* aids in unsupervised learning objective of the generator via adversarial feedback and finally; (6) *auxiliary classifier (AuxC)* assists the generator in learning the relation between input conditionals and their sequences. AE, $$T_r$$ and S undergo pre-training on their respective tasks independently before being jointly trained with G, D and AuxC.

ClinicalGAN consists of six network components, as shown in Fig. 2, that help to achieve the task of conditional data generation. The (a) *auto-encoder (AE)* transforms the data from feature space to latent space; (b) *conditional generator (G)* auto-regressively generates personalized patient journeys; (c) *termination network* ($$T_r)$$ models the drop-off likelihood of patients over their simulated journeys; (d) *conditional discriminator (D)* aids in unsupervised learning objective of the generator via adversarial feedback; (e) *auxiliary classifier (AuxC)* assists the generator in learning the relation between input conditionals and their sequences and finally; (f) *supervisor network (S)* to assist the generator with a supervised objective to further guide the learning process. The key insight in the design is that we transfer the learning process into a lower dimensional learned latent space, such that all the supervised (*AE,S,*$$T_r$$) as well as adversarial (*G,D,AuxC*) components work on latent representations.

Design considerations: The proposed architecture uses Multilayer perceptron (MLP) for modelling static or demographic features, including patient gender, location and other demographics features assumed to be independent and identically distributed (i.i.d). To capture temporal dependencies in time-series features such as lab tests, vitals, Gated Recurrent Units (GRU) are considered due to their ability to capture temporal relationships. This also ensures that the developed model is able to capture cause and symptom relationship that may occur in temporal order.

#### Auto-encoder (AE)

ClinicalGAN uses a sequential auto-encoder (AE)^26,27^ to map the input data from the original data space to a lower dimensional manifold and back. The encoder (Enc) converts the input data into latent space for all the inner learning procedures of the proposed framework. The decoder (Dec) maps the hidden representations back to the original space during the final conditional sequence generation step to produce real-looking data. MedGAN and MedWGAN^28,29^ used auto-encoders for high dimensional cross-sectional medical data compression. The proposed ClinicalGAN extends this concept to spatio-temporal data. ClinicalGAN presents a novel mechanism of not only encoding static conditionals with corresponding sequential journeys to enable maximum information retention in a compressed space, but also facilitate the generator in learning temporal relationships from the data better (discussed in “Proposed architecture: conditional ClinicalGAN”). Specifically, the encoder transforms static and temporal features to their latent representation, i.e., $Enc: \mathcal {X}_s \times \prod _t \mathcal {X}_t \rightarrow \mathcal {H}_s \times \prod _t \mathcal {H}_t$ such that:

3 $$\begin{aligned} h_s = Enc_s(\textbf{s}),& \quad h_t = Enc_t(\textbf{h}_s, \mathbf {h_{t-1}, x_t}), \end{aligned}$$

where, $Enc_s: \mathcal {X}_s \rightarrow \mathcal {H}_s$ is the encoder for static features, $Enc_t: \mathcal {H}_s \times \mathcal {H}_t \times \mathcal {X}_t \rightarrow \mathcal {H}_t$ is the encoder for temporal patient journeys, and $\mathbf {s, x_t}$ are the static and temporal data in original space respectively. Note that static features are encoded independently, however, temporal variables are encoded by auto-regressively conditioning on previous time-step information as well as the corresponding static conditions of the said temporal sequence. Similarly, the decoder reverse maps the learned latent representations to original data space, i.e., $Dec: \mathcal {H}_s \times \prod _t \mathcal {H}_t \rightarrow \mathcal {X}_s \times \prod _t \mathcal {X}_t$ such that:

4 $$\begin{aligned} \tilde{x}_s = Dec_s(\textbf{h}_s),& \quad \tilde{x}_t = Dec_t(\textbf{h}_t), \end{aligned}$$

where, $Dec_s: \mathcal {H}_s \rightarrow \mathcal {X}_s$ is the decoder for static features, and $Dec_t: \mathcal {H}_t \rightarrow \mathcal {X}_t$ is the decoder for temporal patient journeys. We use multi-layer perceptrons (MLPs) as static components of the AE, while GRU^30^ (a form of RNN) as the temporal AE units. Note that these encoders and decoders can be parameterized by any architecture of choice that are auto-regressive in nature that do not violate the causal ordering of information. This causal ordering constraint on the architecture ensures that output at each time-step depends only on the previous time-step information to prevent information leakage. Finally, using static latent vectors as conditional inputs for the temporal encoder facilitates the generator in learning temporal relationships from the data better as we will see subsequently.

#### Termination network ($T_r$)

ClinicalGAN employs a dynamic termination network ($T_r$) to infuse awareness of patient drop-off conditions during the patient journey generation process. Thus, unlike current SOTA TimeGAN, ClinicalGAN is capable of generating different patient-level journey simulations with varying lengths to represent good and bad outcome trajectories under different conditions. This component is a novel contribution of this work as no previous works have used a dynamic termination network in conjugation with a generator to learn the drop-off joint distribution in relation to the evolving patient trajectory. The learning dynamics of this component with others in this architecture is the key to accomplishing the task of conditional patient-level data generation. Specifically, $T_r$ predicts the probability of patients’ drop-off from the trial at each visit based on their journey trajectory so far, such that $T_r: \mathcal {H}_s \times \prod _t \mathcal {H}_t \rightarrow \mathbb {R} \in [0,1]$:

5 $$\begin{aligned} drop\_prob_t = T(\textbf{h}_s, \mathbf {h_{t-1}}) \end{aligned}$$

where, $drop\_prob_t$ is the probability that the patient will drop-off at time step *t*. We parameterize $T_r$ via GRU network. This termination network is dynamic in the sense that it does not make a single prediction of journey length *once* after seeing the entire journey, rather predicts the termination likelihood at each time-step. This equips it to learn the evolution of termination likelihood based on trigger conditions as the journey takes its course. Thus, the proposed dynamic network provides us with much more granular information about the evolution of drop-off probability throughout the various simulation trajectories for further analysis in downstream tasks (e.g., which events increase or decrease the chances of drop-off and how to pro-actively take evasive action accordingly).

#### Conditional generator and discriminator (G and D)

Since we transfer the entire learning process into a lower dimensional manifold, the generator (G) does not produce synthetic samples directly but rather their latent representations. Subsequently, the discriminator (D) is trained to distinguish the latent representations of real data from the generated latent vectors. GANs learn by mapping a known distribution to the learned target distribution via sampling random vectors. Let $\mathbf {Z_t} \in \mathcal {Z}_t$ be the sampled random vector from a known distribution space, then $G: \prod _t \mathcal {Z}_t \rightarrow \prod _t \mathcal {H}_t$ such that:

6 $$\begin{aligned} {\hat{\textbf{h}}}_t = G(\textbf{h}_s, {\hat{\textbf{h}}_{t-1}, z_t}) \end{aligned}$$

where $\textbf{h}_s$ is the latent representation of input static vector obtained from AE component $E_s$ used for conditional generation and $G: \mathcal {H}_s \times \mathcal {H}_t \prod _t \mathcal {Z}_t \rightarrow \mathcal {H}_t$ is the auto-regressive generator of ClinicalGAN which we parameterize with a GRU network. We sample the noise vector $z_t$ from a standard Gaussian distribution using the Wiener process^31^. Subsequently, the discriminator receives the static and temporal representations $D: \mathcal {H}_s \times \prod _t \mathcal {H}_t \rightarrow \mathbb {R} \in [0,1] \times \prod _t \mathbb {R}_t \in [0,1]$:

7 $$\begin{aligned} \tilde{y}_t = D({\tilde{\textbf{h}}}_s, {\tilde{\textbf{h}}^*}_t), \end{aligned}$$

where ${\tilde{\textbf{h}}^*}_t$ notation indicates either real ($\textbf{h}_t$) or generated (${\hat{\textbf{h}}}_t$) temporal latent vectors. While the G network is auto-regressive in nature, the D network is not bound by such causal ordering constraints. This is because the G can only auto-regressively generate future steps information based on past context. However, D’s task is to provide feedback to G about how realistic is the entire generated sequence. Thus, the D has the entire sequence available to accumulate left (past) as well as right (future) context into its predictions at each time step, which gives more informative feedback to the generator to improve its generation. To that end, we parameterize D with a bi-directional GRU with MLP classification layer, i.e., two parallel GRU units that take the sequences left-to-right and reverse respectively as input to make the final classification.

Furthermore, due to the auto-regressive design of the conditional generator, ClinicalGAN possesses two types of generation capability depending on the use-case. The first is *Type A* generation wherein it generates the entire patient journeys from scratch using just their conditional vectors. This type of generation capability can be leveraged when creating synthetic private versions of real datasets, where the entire data needs to be replicated but with privacy guarantees preventing patient re-identification. The second is *Type B* generation, where ClinicalGAN can be leveraged to simulate/impute the remainder of the journey when some partial time-steps information is available along with the conditional variables. Such generation capability allows ClinicalGAN to be deployed in trial monitoring scenarios because as more historical time step information of patient visits is made available, their future journey trajectories are adjusted to be simulated with greater precision. Thus, ClinicalGAN with its auto-regressive conditional generator can be effectively deployed in a host of different real-world scenarios depending on the kind of inputs available.

#### Auxiliary classifier (AuxC)

The aim of ClinicalGAN is not to just produce real-data-like patient journeys, but to condition the simulations on specified patient-level conditional features (demographics, medical history, etc). Since the aforementioned discriminator is designed to just discriminate between real and generated sequences, it does not explicitly steer the generator to generate coherent sequences while adhering to the static conditionals. This leads to mode collapse during generation. Mode collapse is the phenomenon where the generator outputs a similar set of sequences irrespective of the input conditional vectors since it has not learned the relationship between these conditional vectors and the respective generated sequences. To tackle this issue, we employ the auxiliary classifier (AuxC) to assist the generator in learning the joint distribution $\hat{p}({\hat{\textbf{X}}}_t | {\hat{\textbf{X}}}_s, {\hat{\textbf{X}}}_{1:t-1})$ much more effectively. Specifically, AuxC works in reverse nature of the generator objective such that it predicts the input conditional vector given a sequence of latent journey representations such that, $AuxC: \prod _t \mathcal {H}_t \rightarrow \mathcal {H}_s$:

8 $$\begin{aligned} \textbf{H}_s = AuxC({\tilde{\textbf{h}}^*}_{T}) \end{aligned}$$

where $({\tilde{\textbf{h}}^*}_{T})$ denotes either real ($\textbf{h}_{T}$) or generated (${\hat{\textbf{h}}}_{T}$) final step latent representation of patient journeys. We parameterize AuxC with a MLP layer. The concept of auxiliary-classifiers has existed in image synthesis literature^32,33^, but no work has been done to adopt this technique for the spatio-temporal setting. This work introduces a novel auxiliary classifier setup for sequential auto-regressive architectures to demonstrate how this technique can be used to improve the generation quality of patient-level medical data. The AuxC introduced in ClinicalGAN achieves the task of explicitly connecting parts of the latent space to model inputs, thereby establishing a direct conditional relation between the *given input* and the *expected output*. Unlike image synthesis where the input is a specific class (categorical input) referring to the object in the image, the input condition in this case is a multi-variate conditional vector mapped into the latent space. Thus, the AuxC component here has to learn more complex relations between the output sequence and the complex input vector.

#### Supervisor network (S)

Pure unsupervised adversarial loss (from D and AuxC) may not be enough for the generator to learn the spatio-temporal relations efficiently. Thus, we introduce the supervisor network (S) to further discipline the training and assist the generator with a direct supervised loss for explicit feedback in its auto-regressive generation process. Specifically, $S: \mathcal {H}_s \times \prod _t \mathcal {H}_t \rightarrow \mathcal {H}_t$, such that:

9 $$\begin{aligned} \textbf{h}_{t} = S(\textbf{h}_s, \mathbf {h_{t-1}}) \end{aligned}$$

Thus, the role of the supervisor network is to input information at time step $t-1$ to produce data at *t*. This is leveraged during the generator training phase where generated sequences are fed to the supervisor to compute supervised loss on the generator’s outputs. We parameterize S with a GRU network.

### Multi-objective training

All the components of ClinicalGAN are trained using semi-supervised as well as unsupervised learning paradigms. As shown in Fig. 2, we split the training pipeline into two steps. The first step is to pre-train the individual components of ClinicalGAN on their respective objectives to infuse them with a preliminary knowledge of their tasks. The second step is to jointly train all the networks together to align their latent spaces into a common manifold to achieve the overall conditional generation objective.

The auto-encoder is trained to create a mapping between the feature and latent spaces, and thus should be able to accurately reconstruct $\tilde{x}_s and \tilde{x}_{1:T}$ of original $x_s, x_{1:T}$ from the latent representations $h_s, h_{1:T}$. This is achieved by training AE on reconstruction loss, which is realized by mean squared error (MSE), such that:

10 $$\begin{aligned} \mathcal {L}_R = \mathbb {E}_{x_s, x_{1:T} \sim p} \Bigr [||x_s - \tilde{x}_s ||_2 + \sum _t ||x_t - \tilde{x}_t||_2 \Bigl ] \end{aligned}$$

The termination network is trained to predict the probability of the patient drop-off from the trial at each time step based on its past context $h_s, h_{t-1}$. This is achieved by training the T on drop-off prediction loss, realized by binary cross entropy (BCE), such that:

11 $$\begin{aligned} \mathcal {L}_{T_r} = \mathbb {E}_{x_s, x_{1:T} \sim p}\Bigr [-w_n [ y_n \cdot \text {log} \sigma (x_n)+ (1 - y_n) \cdot \text {log}(1 - \sigma (x_n))\Bigl ] \end{aligned}$$

where *n* denotes the mini-batch size and $w_n$ denotes the weight for positive class to include when dealing with unbalanced label distribution. Since “termination event” is only at the end of the sequence with majority of the sequence having the label “continues”, we note a high degree of class imbalance in this binary classification task. Thus, we employ positive class re-weighting using $w_n$ to theoretically balance the weights for each class. Furthermore, in our experiments, we tackle the drop-off prediction as a binary classification task, due to the unavailability of drop-off reason in the datasets used. However, the above task can be easily extended to a generalized setting of multi-class classification setup as well, where the $T_r$ network predicts whether the patient is likely to continue or drop-off due to one of the *C* reasons at each time-step. In this case, the loss formulation changes to a cross-entropy (CE) loss, such that:

12 $$\begin{aligned} \mathcal {L}_{T_r} = - \sum \limits _{c=1}^C w_c \cdot \text {log} \Bigg (\frac{\text {exp}(x_{n,c})}{\sum \limits _{i=1}^C \text {exp}(x_{n,i}) } \Bigg ) \cdot y_{n,c} \end{aligned}$$

where *x* is the input, *y* is the target, $w_c$ is the per class weight, *C* is the total no. of classes and *n* is the mini-batch dimension. Due to the limitation of the information available in the datasets used, we pose the evaluation of this generalized setting for the task of drop-off prediction as future work.

The original GAN training objective is designed to predict how real an input is (*D*(*x*)). However, this objective suffers from vanishing gradients problem early on in the training. Thus, we employ the relativistic GAN (RSGAN) objective^34^ to tackle this issue and stabilize GAN training. The RSGAN computes a “distance” $D(x_r, x_f)$, i.e., the probability that the real data is more realistic than the fake data which gives us the unsupervised adversarial objective to be:

13 $$\begin{aligned} \mathcal {L}_U&= L_D^{RSGAN} + L_G^{RSGAN}\end{aligned}$$

14 $$\begin{aligned} \mathcal {L}_U&= -\mathbb {E}_{(h_s, h_{1:T} \sim p) \text { ; } (\tilde{h}_s, \tilde{h}_{1:T} \sim \tilde{p})} \Big [\sum _t \text {log} (\sigma (y_t - \tilde{y}_t) + \sum _t \text {log} (\sigma (\tilde{y}_t - y_t) \Big ] \end{aligned}$$

As mentioned in “Conditional generator and discriminator (G and D)”, ClinicalGAN is capable of *Type A* and *Type B* forms of generation scenarios based on the real-world use-cases. We incorporate this capability in the training dynamics as well, by training the auto-regressive conditional generator in closed-loop (generative) mode for *Type A* generation and open-loop (teacher forcing) mode for *Type B* generation. The open and closed loop training of the generator are distinct from one another in a simple way that the previous step input to the generator comes either from self-generated information or from the ground truth, i.e., real data. In the open loop mode, the generator is recursively fed with temporal information from its own generation of the previous step, i.e., $p(\textbf{H}_t | \textbf{H}_s, \textbf{H}_{1:t-1})$ is approximated by $\hat{p}({\hat{\textbf{H}}}_t | \textbf{H}_s, {\hat{\textbf{H}}}_{1:t-1})$. Meanwhile, in the open loop mode, the generator is fed with partial sequences of real data journey at each step, i.e., $p(\textbf{H}_t | \textbf{H}_s, \textbf{H}_{1:t-1})$ is approximated by $\hat{p}({\hat{\textbf{H}}}_t | \textbf{H}_s, \textbf{H}_{1:t-1})$. The unsupervised loss function remains the same post this subtle change in the generation process.

Subsequently, to assist the conditional generator in better learning the dependencies between the input conditional vectors and the resultant journeys output, we employ the auxiliary classifier (AuxC). The AuxC network is trained to predict the original conditional latent vector (coming from real data) given the patient journey. This is achieved by training the AuxC on reconstruction loss realized via MSE such that:

15 $$\begin{aligned} \mathcal {L}_{AuxC} = \mathbb {E}_{(h_{T} \sim p) \text { ; } (\hat{h}_{T} \sim \tilde{p})} \Bigr [||h_T - \hat{h}_T ||_2 \Bigl ] \end{aligned}$$

Finally, to assist the generator with an additional supervised loss, the supervisor network takes the inputs of the generator to predict the real next-time-step information. The gradients are thus computed on MSE supervised loss that measures the discrepancy between the distributions $p(\textbf{H}_t | \textbf{H}_s, \textbf{H}_{1:t-1})$ and $\hat{p}({\hat{\textbf{H}}}_t | \textbf{H}_s, {\hat{\textbf{H}}}_{1:t-1})$ such that:

16 $$\begin{aligned} \mathcal {L}_S = \mathbb {E}_{x_s, x_{1:T} \sim p} \Bigr [\sum _t ||h_t - G(h_s, h_{t-1}, z_t) ||_2 \Bigl ] \end{aligned}$$

where $G(h_s, h_{t-1}, z_t)$ approximates $\mathbb {E}_{z_t \sim \mathcal {N}}[\hat{p}({\hat{\textbf{H}}}_t | \textbf{H}_s, {\hat{\textbf{H}}}_{1:t-1})]$. In essence, during the training phase, ClinicalGAN computes the difference between the latent embeddings of real data (from the encoder) and the generated latent vectors (from the generator) while being conditioned on the real patient meta-data latent vectors. The task of the generator is to auto-regressively generate next-step information that resembles the real data by conditioning on historical temporal information as well as the conditional vectors. This task is achieved by disciplining the generator via the unsupervised ($\mathcal {L}_U$) as well as supervised losses ($\mathcal {L}_S$). While $\mathcal {L}_U$ assists the generator in learning to produce realistic spatio-temporal sequences (assessed by an imperfect adversary in a minmax game), the $\mathcal {L}_S$ pushes the generator to be consistent in its step-wise auto-regressive generation.

Optimization The first phase of ClinicalGAN training involves pre-training all the supervised components (AE, T, S) on their respective objectives as a warm-up. In the second phase, both the supervised and unsupervised components are trained jointly under various unified objectives as detailed below.

Let $\theta _{enc}, \theta _{dec}, \theta _{T}, \theta _{S}, \theta _{G}, \theta _{D}, \theta _{AuxC}$ denote the parameters of encoder, decoder, termination network, supervisor, generator, discriminator and auxiliary classifier respectively. Then AE components are trained on the reconstruction as well as the supervised loss while the termination network is trained solely on its supervised cross entropy loss such that:

17 $$\begin{aligned} \underset{\theta _{enc}, \theta _{dec}}{min} (\lambda \mathcal {L}_S + \mathcal {L}_R )  ;& \quad \underset{\theta _{T}}{min} \mathcal {L}_T \end{aligned}$$

where, $\lambda \ge 0$ is the relative weight of supervised loss in the objective. Note that the encoder is trained on the supervised loss as well since it is tasked with not just creating low dimensional latent embeddings, but also assisting the generator to learn the spatio-temporal relationships better. Next, since the termination network is trained to predict the drop-off dynamically in the auto-regressive process, it is fine-tuned as mentioned above. The joint training is based on the updated input space coming from AE and G components, since they are brought into a common latent manifold during this phase of training. Finally, the conditional generator and discriminator with auxiliary classifier are trained adversarially as:

18 $$\begin{aligned} \underset{\theta _{G}}{min} \Big (\eta \mathcal {L}_S + \underset{\theta _{D}, \theta _{AuxC}}{max} (\mathcal {L}_U + \delta \mathcal {L}_{AuxC}) \Big ) \end{aligned}$$

where, $\eta , \delta \ge 0$ are the relative weights of supervised and auxiliary loss respectively. This form of multi-objective training equips ClinicalGAN to encode feature vectors into a joint latent space, generate latent representations in the same space and dynamically predict patient drop-off as each journey progresses—all while conditioning the entire process on patient meta-data for a personalized generation.

During our experiments we note that ClinicalGAN is not sensitive to $\lambda , \eta \text { and } \delta$, as the training dynamics do not vary substantially based on the relative re-weighting of the respective losses. We thereby set them to values that bring respective losses into similar value ranges (such as $10^{-2}$) since different losses of the multi-objective training can have different magnitudes. Furthermore, GANs are typically known to be unstable during training suffering from problems like vanishing gradients and mode collapse. However, ClinicalGAN is specifically designed to avoid these pitfalls. While using the RSGAN objective helps to tackle the vanishing gradients problem, auxiliary classifier along with supervised training help in preventing mode collapse. Thus, special care has been taken to ensure that training ClinicalGAN is not expected to be more challenging than other generative methods.

## Results and analysis

### Data

For CODR-AD, we extracted complete longitudinal trajectories of 1130 patients including 21 variables consisting of ADAS-Cog and MMSE scores, laboratory tests, and background information of patients. The second data was extracted from Data Usage Agreement for the Alzheimer’s Disease Neuroimaging Initiative (ADNI) [Data used in the preparation of this article were obtained from the Alzheimer’s Disease Neuroimaging Initiative (ADNI) database (adni.loni.usc.edu). As such, the investigators within the ADNI contributed to the design and implementation of ADNI and/or provided data but did not participate in analysis or writing of this report. A complete listing of ADNI investigators can be found at this document]. We used the publicly available version of this dataset available as part of Kaggle competition—ADNI public dataset. This data contains complete longitudinal trajectories of 971 patients including 14 variables consisting of similar information as the CODR-AD dataset. Table 2 summarizes some basic data statistics of the final datasets used after pre-processing. The details of pre-processing for both the datasets are provided in the Supporting Information.

**Table 2:** Summary statistics of CODAR-AD and ADNI datasets.

Dataset	No.of patients		Sequence length			Feature type		
	Train	Test	Min.	Max.	Avg.	Continuous	Categorical	Ordinal
CODR-AD	1127	197	3	12	7.14	12	5	4
ADNI	770	201	2	10	4.82	8	3	3

### Computation considerations

We ran all the experiments for this work on Amazon AWS cloud using the G4dn instances. G4dn instances are the lowest cost most basic GPU-based instances in the cloud for machine learning inference and small scale training. G4dn infrastructure details are mentioned in Table 3 below:

**Table 3:** Infrastructure details of the Amazon AWS cloud G4dn instance used in all ClinicalGAN experiments in this work.

Compute attribute	Value
vCPUs	8
Memory (GB)	32 GB
Physical processor	Intel Xeon family
Clock speed (GHz)	2.5 GHz
No. of GPUs	1
GPU architecture	Nvidia T4 tensor core
GPU memory (GB)	16 GB (14.8 GB usable)

Although it is a multi-component architecture, we have kept the data size and compute availability in mind for its practical application. We have designed it specifically to work well in low data scenarios with a very light computation footprint. The architecture does not require large amounts of data and high compute resources to perform well as compared other transformer architectures. For instance, although the GPU instance used had 15GB available memory, ClinicalGAN even in its highest configuration tested never utilized more than 3GB of GPU. In Table 4 we provide more details of the training and inference times.

**Table 4:** Training (pre-training + joint) and inference times of ClinicalGAN. *sim* refers to individual patient level simulations.

Attribute	Value
Training time calculation	
No. of epochs (pre-training components)	2500 (each for AE, S and $T_r$)
No. of epochs (joint components)	10,000
Training time (Total)	$\sim 5.5$ h
Training time (epochs/s)	$\sim 0.9$ epochs/s
Inference time calculation	
No. of unique patients to be simulated (test)	250
No. of simulations per patient	1000
Inference time (total)	158 s
Inference time (ms/simulations)	$\sim 0.632$ ms/sim
Inference time (simulations/s)	$\sim 1585$ sims/s

We can see that even with a multi-component architecture and running on smallest available instance, ClinicalGAN still trains at almost $\sim 1$ epoch/s while inference is also super efficient at $\sim 1600$ simulations/secs. This shows that the architecture can be trained and used on simple compute resources making it very practical for real-world scenarios.

Hyper-parameter tuning: The hyper-parameters used in ClinicalGAN are standard across any typical deep learning framework, specifically GANs. Thus, there is no additional effort involved in training this framework as opposed to any other SOTA deep learning models. Secondly, to limit the hyper-parameter search space further and speed up the training phase, we have employed many known best-practices while training GANs based on existing literature in this domain (summarized here for instance: training GANs guide). The hyper-parameters in this reduced search space were then optimized using random search using a validation set.

### Benchmarks and evaluation

**Benchmarks** While there are a few recent advanced works such as RC-GAN^7^, C-RNN-GAN^6^ and TimeGAN^5^ for the task of multi-variate timeseries generation, TimeGAN is the current state-of-the-art (SOTA) for the task. Additionally, as seen from Table 1, TimeGAN comes the closest to our proposed framework in terms of required capabilities for the task of clinical trial data generation. Furthermore, the authors of TimeGAN have extensively benchmarked it with the aforementioned methods and shown it to outperform all of them to be the current SOTA. Thus, we benchmark the performance of ClinicalGAN against the current SOTA TimeGAN in this work. Additionally, as part of the ablation study to isolate the sources of performance gain of the proposed architecture, we introduce two versions of ClinicalGAN for comparison—*CGAN (−AuxC)* and *CGAN (+AuxC)*. The two architectures differ in solely one aspect, i.e., *CGAN (+AuxC)* has discriminator+AuxC network to steer the conditional generation better, while *CGAN (−AuxC)* is equipped with just the discriminator network for adversarial feedback. Note that all the remaining components are part of the basic structure of the proposed architecture for the given task, with AuxC being the only optional unit added to improve conditional generation. Further, to prevent information leakage and mimic real-world scenarios of working on unseen data, we make train and test splits (80% train and 20% test) of the data and train the generative models *only* on the train set. The generative models are then tested on the unseen test set over 1000 simulations for each patient in the test set, to assess their generalizing capability to unseen data. All the subsequent analysis from hereon for all the models is based on the held-out test set of both the datasets. We report all the performance scores for *CGAN (−AuxC)* and *CGAN (+AuxC)* variants along with the TimeGAN baseline in Table 5. This demonstrates the contribution of the AuxC network in the architecture design, and serves as an ablation experiment to isolate sources of performance gain of ClinicalGAN. However, for all the visual depictions (wherever applicable) we present the performance of just *CGAN (+AuxC)* variant (referred to as *ClinicalGAN* hereon), since it is the best performing variant of the proposed ClinicalGAN framework.

**Table 5:** Generation quality metrics results for CODR-AD and ADNI datasets over 1000 simulations for each patient in the test set.

Dataset	Model name	ACD $\downarrow$	FID $\downarrow$	Alpha-PR $\uparrow$	Discriminator score $\downarrow$			Next-step prediction $\downarrow$	
					AUCROC	PRAUC	F1	Real MSE	Gen MSE
CODR-AD	TimeGAN^5^	0.557	7.03	0.02	0.881	0.913	0.821	0.013	0.099
	CGAN (−AuxC)	0.294	2.58	0.52	0.714	0.623	0.736	0.013	0.048
	CGAN (+AuxC)	**0.246**	**0.87**	**0.72**	**0.515**	**0.535**	**0.674**	0.013	**0.023**
ADNI	TimeGAN^5^	0.558	7.37	0.02	0.99	0.99	0.99	0.015	0.16
	CGAN (−AuxC)	0.271	2.54	0.46	0.80	0.72	0.82	0.015	0.045
	CGAN (+AuxC)	**0.202**	**1.07**	**0.77**	**0.77**	**0.68**	**0.77**	0.015	**0.027**

ACD represents the $Avg\_corr\_diff$ scores. Values in Bold represent best scores and in Underline represent second best scores within a dataset. $\uparrow$ denote higher scores are better for that metric, and $\downarrow$ the vice versa.

**Evaluation** We holistically evaluate the aforementioned models on three dimensions of evaluations that represent desirable generation quality—(a) *fidelity*, (b) *diversity* and, (c) *utility*.

#### Generation fidelity

**Figure 3:**
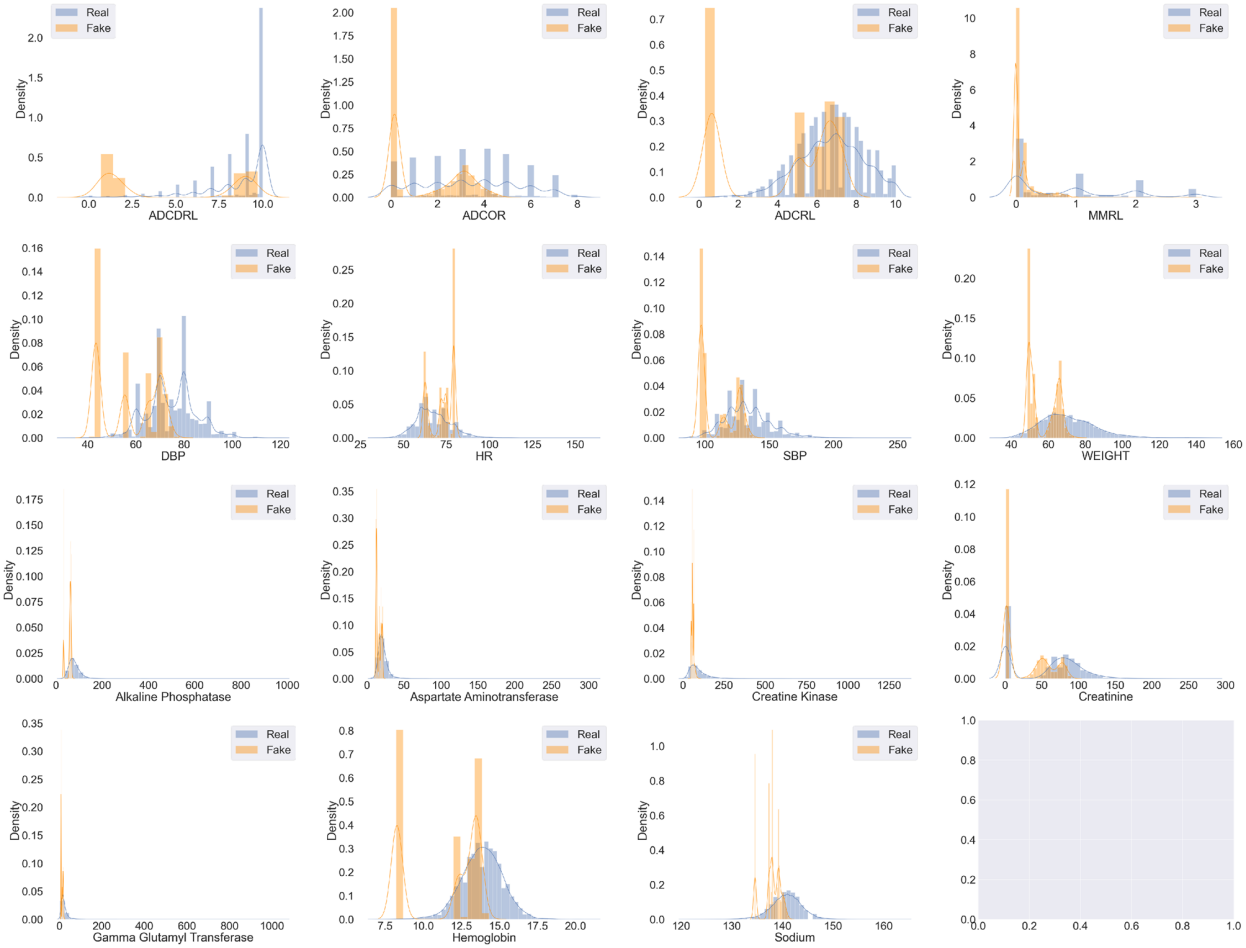
KDEs of TimeGAN on CODR-AD dataset.

The fidelity of generated data entails how coherent are the generated journeys in terms of inter-variate correlations and adherence to patient meta-data. These metrics measure how well has the model learned the underlying data distribution, resulting in generated samples being indistinguishable from real ones. We use 5 fidelity metrics:

1. **Univariate KDEs:** we begin our evaluation with qualitative univariate evaluation to assess if the model can capture the various modes of each of the mixed-type variables well. We use kernel density estimate (KDE) plots to visualize the multi-modal distributions of variables in both the datasets. Figures 3 and 4 show the KDE plots for TimeGAN and ClinicalGAN on CODR-AD dataset respectively over 1000 simulations for each patient in the test set. We observe that the mean as well as the variance across all the variables is much more accurately modeled by ClinicalGAN than TimeGAN. ClinicalGAN shows to consistently capture all the modes including the long tail of the distributions quite well, which is typically difficult to model in low resource settings and becomes a failure case for most AI/ML models. On the other hand, TimeGAN tends to focus its generation just around the mean of one of the modes and fails to capture the breadth of variance of each variable effectively. KDEs for both the models on the ADNI dataset also demonstrate the same trends in performance and can be found in the Supplementary Figs. 11 and 12.

**Figure 4:**
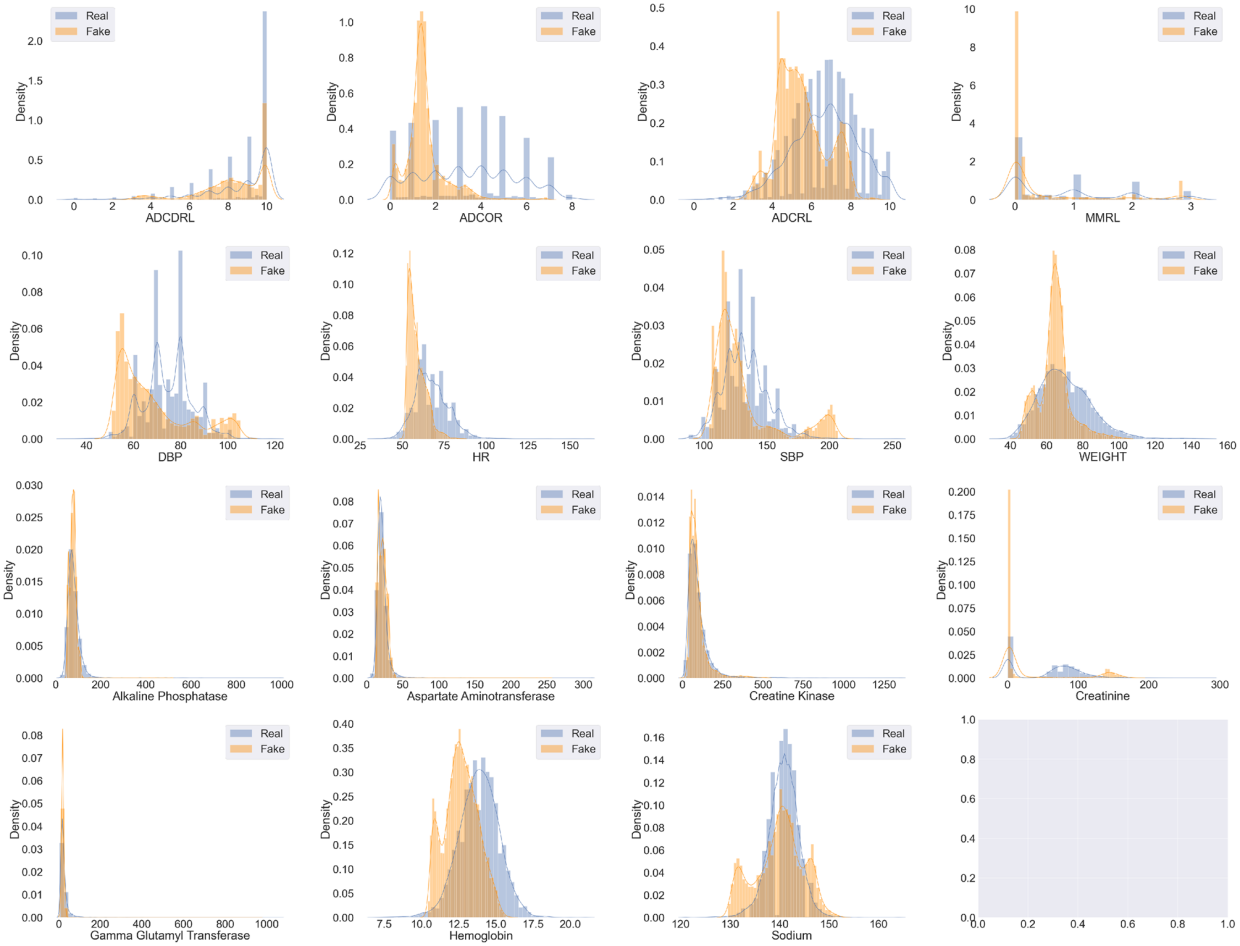
KDEs of ClinicalGAN on CODR-AD dataset.
2. **Correlation heatmaps:** next, we assess how well the model captures the bivariate correlations between these variables of underlying real data. Since we are dealing with mixed-type data, we employ multiple correlation strategies for different type pairs:
  *Continuous–continuous*: Pearson’s correlation^35^. It can measure correlations while being robust to outliers as opposed to Spearman’s correlation metric. It essentially measures how does the value of given a continuous variable vary in relation to another continuous value.
  *Continuous–categorical*: Correlation ratio^35^. It measures the weighted variance of the mean of each category divided by the variance of all samples. It essentially measures how well can we predict the category a continuous value belongs to.
  *Categorical–categorical*: Theil’s U^36^. It is based on the conditional entropy between two variables also known as Uncertainty Coefficient. It essentially measures how many values and with what probabilities there are for variable *x*, given a value for variable *y*.
  To quantify the quality of correlations captured, we define aggregated correlation as the mean value of all the absolute bi-variate correlations from the upper triangular matrix. Specifically,
  19 $$\begin{aligned} Avg\_corr\_diff = \frac{1}{p} \sum _{i,j} |corr_R[f_i, f_j] - corr_G[f_i, f_j]| \end{aligned}$$
  where *p* denotes the total no. of bi-variate feature combinations, *i*, *j* denote the pair of features, and $corr_*[f_i, f_j]$ denotes the correlation function (one of the three mentioned above) depending on the feature type of *i*, *j* of real or generated data. Lower value of $Avg\_corr\_diff$ indicates similar correlations captured in real and generated data, and thus better generation. In Fig. 5 we visualize the bivariate correlation heatmaps for the (a) original data, (b) generated data of TimeGAN and ClinicalGAN, as well as (c) the difference between the real and generated correlations for each model over 1000 simulations for each patient in the test set. Lighter shades of difference plots indicate lower differences between real and generated data correlations and better correlation captured by the model. We clearly note that ClinicalGAN captures the underlying correlations much more closely than TimeGAN, with a higher ratio of lighter shades in the difference plots. This is also reflected in the quantified $Avg\_corr\_diff$ metric as reported in Table 5 with ClinicalGAN being 2.5x better than TimeGAN on average in capturing bivariate correlations. Correlation heatmaps for both the models on the ADNI dataset also demonstrate the same trends in performance and can be found in the Supplementary Fig. 13.

**Figure 5:**
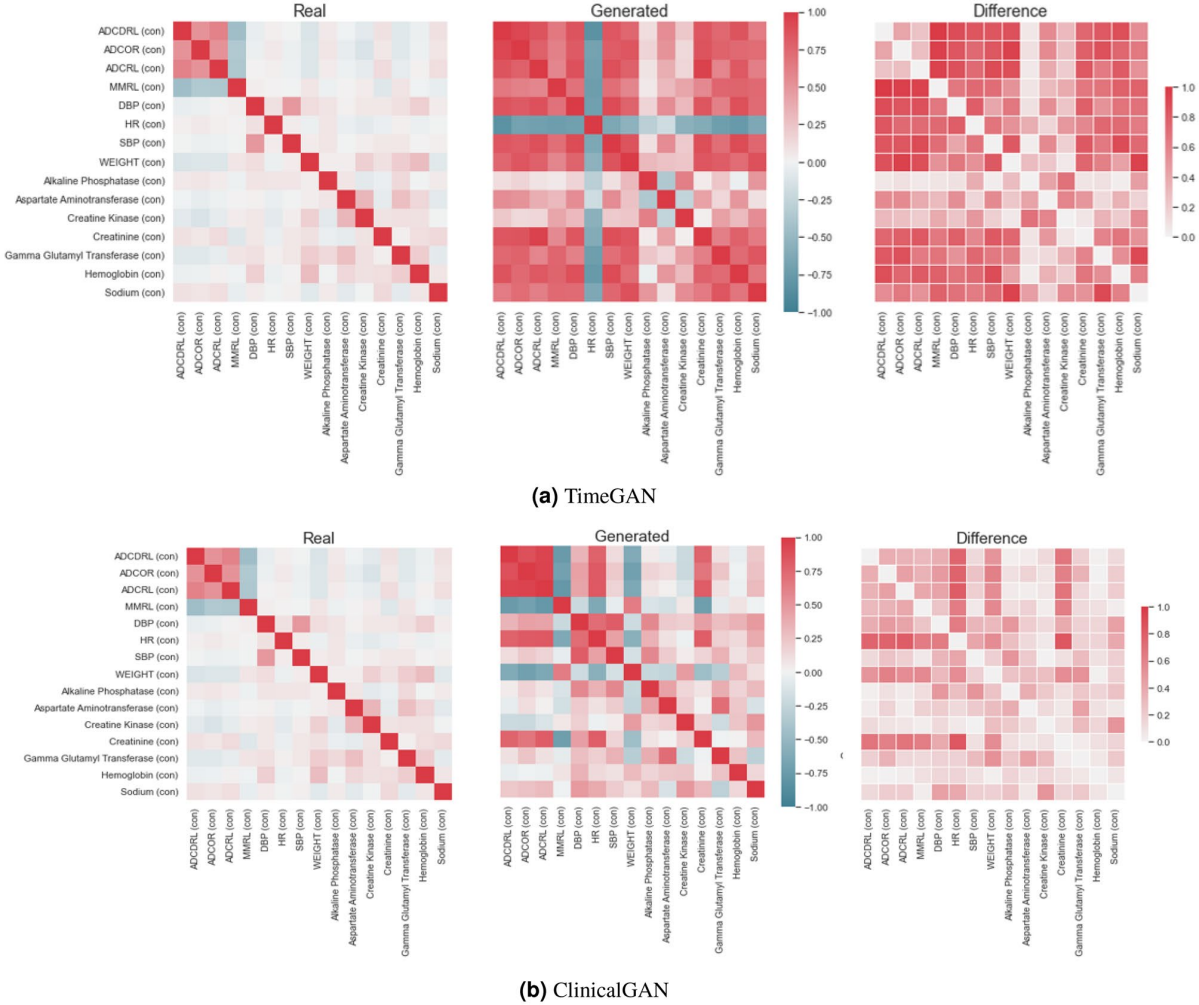
Correlation heatmaps of ClinicalGAN and TimeGAN models on CODR-AD dataset.
3. **t-Distributed stochastic neighbor embedding (tSNE)**^37^: is used to visualize high-dimensional data that lie on several different, but related, low-dimensional manifolds (such as journeys of patients from multiple cohorts), as seen from multiple viewpoints. We visualize the real and generated data embeddings in a common 2D space to assess how close do the journeys lie in the latent space for the same set of patients’ meta-data. The sequence embeddings of real and generated patient journeys represent their sequence representations in a latent space and are obtained from the common sequential encoder network of ClinicalGAN. These *n*-dimensional embeddings are then projected onto a 2-dimensional space using the tSNE method. In Fig. 6, each red point represents the 2-D projection of a patient’s clinical journey, while blue points represent generated sequences in this projection space over 1000 simulations for each patient in the test set. We can clearly see that ClinicalGAN captures multiple regions of data well, exhibiting better diversity across both the datasets. On the other hand, TimeGAN exhibits mode collapse and results in generating similar journeys around a small region of latent space in both the datasets. This demonstrates that ClinicalGAN generalizes much better to unseen data due to its conditional generation capability. The conditional training equips ClinicalGAN with interpolation and extrapolation ability and allows it to retain a stronger prior (learned from jointly modeling $\mathbf {h_s}$ during training) when generating data for unseen distribution.

**Figure 6:**
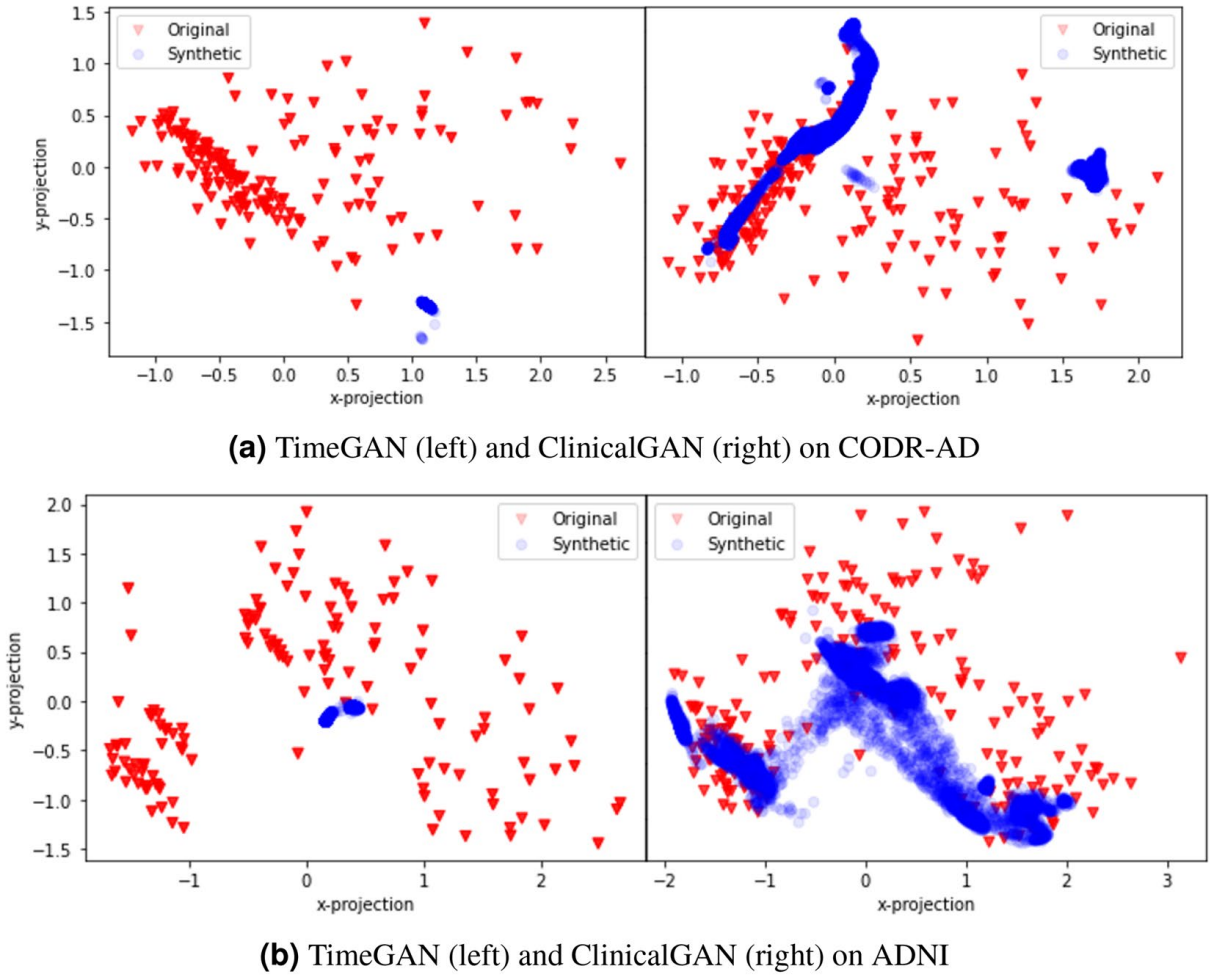
tSNE plots of latent representations of proposed CGAN (+AuxC) and baseline TimeGAN on CODR-AD (left) and ADNI (right) datasets. Each red (real data) and blue (generated data) points represent the 2D projection of 128-dim latent representation of a patient’s journey.
4. **Frechet Inception Distance (FID)**^38^: is a measure of similarity between two distributions while also taking the multi-variate correlation between features into account such that:
  20 $$\begin{aligned} FID_{RG} = ||\mu _R - \mu _G||^2 - Tr(\Sigma _R + \Sigma _G - 2 \sqrt{\Sigma _R \Sigma _G}) \end{aligned}$$
  where R and G are the real and generated data embeddings, $\mu _R$ and $\mu _G$ are the magnitudes of R and G, Tr is the trace of matrix and $\Sigma _R$ and $\Sigma _G$ are the covariance matrices of the vectors. FID measures how well are the spatio-temporal correlations of real data preserved in the generated data. To measure FID, we obtain the embedding of a sequence as the final hidden state of the common encoder RNN of the ClinicalGAN auto-encoder network. FID is bounded between $[0, \inf ]$ and since it is a distance measure, a lower FID score implies smaller distances between real and generated samples, thereby indicating better generation quality. As seen from Table 5, ClinicalGAN significantly outperforms TimeGAN on FID scores across both the datasets, indicating superior spatio-temporal trend capture capability.
5. **Discriminator score (D-score)**: is the classification performance of a sequence classifier in distinguishing real samples from generated ones. We report F1, PrAUC, AUCROC as the standard balanced binary classification task metrics. Scores closer to 0.5 denote that the classifier can not tell real samples from generated ones, thus implying better generation. As seen from Table 5, ClinicalGAN succeeds in generating indistinguishable simulated journeys of patients with scores closer to 0.5 attesting to its superior conditional generation performance. Meanwhile, TimeGAN fails at generating journeys that look similar to real patient trajectories across both the datasets as the sequence classifier can easily distinguish its generated data from real.

#### Generation diversity

Deep generative models, especially GANs, are susceptible to the phenomenon of mode collapse (generate the same journey repeatedly, but generate it really well). Thus, the generation diversity metric evaluates how well can multiple types of journeys present in the data being generated. We use alpha-precision score as a measure of the overlap of the real and generated data in the latent space to evaluate the diversity of generated samples.

6. **Alpha-precision (Alpha-Pr)**^39^: is the fraction of synthetic samples that resemble the most typical fraction $\alpha$ of real samples. It measures the likelihood of a private sample $y_g$ belonging to the real distribution $\mathcal {D}_{Real}$ within its $\alpha$-support (e.g. a random sub-set of the real data, determined by $\alpha$-mass). Higher scores imply better distribution overlap between real and generated data indicating the model captures all the modes of $\mathcal {D}_{Real}$. We report alpha-Pr scores for $\alpha = 0.95$ in Table 5. We observe that ClinicalGAN covers 72% and 77% of the real data for CODR-AD and ADNI datasets respectively, while TimeGAN manages to cover just 2% of the real data diversity. The TimeGAN architecture thus suffers severely from mode collapse, while ClinicalGAN successfully navigates this issue owing to its architecture design.

#### Generation utility

The generated data should match the performance of real data at downstream ML tasks to demonstrate its fidelity in real-world use-cases. This aspect is captured by the generation utility metric which can be any downstream ML task. We use next time-step prediction as a common ML task to assess the utility of generated samples against real data.

7. **Next-step prediction (NxsP):** to evaluate the utility of the generated journeys, we perform a downstream ML-task of next time-step prediction where we use historical data $[{\textbf {X}}_s, {\textbf {X}}_{1:t-1}]$ to predict all the time dependent variables at the next time-step ${\textbf {X}}_t$. We use independent post-hoc GRU networks for this task that take the previous step information (from real and generated sequences) as input and output the multi-variate prediction of the variables in the next time step. The idea is that there should be minimal difference in performance on a common unseen real data test set between two models that are trained on real and generated data independently but with the same hyper-parameters. We thereby train NxsP models $NxsP_R$ and $NxsP_G$ on real train set $D_{Rtrain}$ and generated data $D_{Gtrain}$, and finally test the performance of both the models on the common real test set, $D_{test}$. From Table 5 we can see that ClinicalGAN again significantly outperforms TimeGAN by having performance closest to the models trained on real data. This demonstrates that generated data from ClinicalGAN very closely replicates the properties of real data, while TimeGAN fails to do so. Thus, data generated via ClinicalGAN can be used as a close substitute to the real data for any analytics use cases where data needs to be shared with a third party while preserving the privacy of the participants.

#### Generation accuracy over varying look-ahead horizons

**Figure 7:**
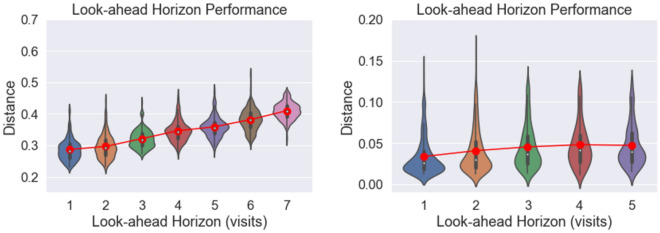
Simulation performance over varying look-ahead steps for CODR-AD (left) and ADNI (right) datasets. The violin plots denote the variance in intra-step JSD distances between real and generated samples, while the red line denotes the mean JSD distance at each step.

ClinicalGAN models the spatio-temporal relationships of multiple variables jointly over variable length journeys. In such cases, it is crucial to assess the quality of generation at time-steps farther into the future. This helps to establish the reliability of the generation at various look-ahead horizons, since the forecasting accuracy tends to deteriorate over longer distances mainly due to error accumulation. To evaluate ClinicalGAN’s performance at long-term trend capture, we design an experiment as follows. Let $x_i = \{x_1, x_2, x_3,..., x_t\}$ be the actual sequence of observations of a particular patient from real-data having *t* time-steps information where $x_i$ represents the multi-variate information at time-step *i*. We then simulate probable paths for that patient using ClinicalGAN for multiple look-ahead horizons such that $y_{j>i} = \{y_{j+1}, y_{j+2},..., y_{t}\} \text { }; \forall i \in [1,t-1]$ by feeding the first *i* real time-steps information as input to the generator and simulating the remaining steps information. We repeat this exercise for multiple simulations for each patient to obtain a distribution of real data values at each time-step as well as of corresponding simulated values for each look-ahead horizon. For instance, we predict the value of actual data at step = 5, by having various degrees of prior information available, i.e., predict step 5 auto-regressively using just step 1 real information (implying a four step look-ahead prediction). Similarly, the same step 5 can be assessed as a 3-step-ahead prediction by feeding the generator with the first 2 steps of real data and auto-regressively predicting step 5; and so on for all look-ahead steps 1, 2, 3, 4. We then calculate the distance between the real and generated samples at each temporal step to evaluate how close do the generated and real samples lie at each horizon window. We report the results with standard multivariate Jensson–Shannon distance ($JSD \in [0,1]$ ; lower the better) as the distance measure in our experiments, but we note that the shown model performance is consistent irrespective of the choice of distance metric used to evaluate. Figure 7 depicts the distribution of these distances over 1000 simulations for CODR-AD (left) and ADNI (right) datasets respectively. We analyze the performance over 7 steps for CODR-AD and 5 for ADNI as these are the median lengths of their respective patient journeys. The no. of patients with sequence lengths more than the median falls drastically to below 20% post the specified time steps. We can see that as the look-ahead horizon increases (i.e. we try to predict farther time-steps with less historical data available as input), the distances between real and generated samples increase slightly. This indicates that ClinicalGAN’s simulation accuracy over a longer horizon does drop progressively. However, it is important to note that the drop in performance is not substantial and the model is able to retain much of its predictive performance fairly well (for instance, 0.28 at step 1 vs 0.38 at step 7 for CODR-AD) over the long-term prediction window. This demonstrates that ClinicalGAN’s design enables it to model and generate long-term correlations in the data consistently. The deterioration in performance for ADNI is even less severe over the projected time steps. Overall, we note that ClinicalGAN exhibits good intra-step performance (good correlation capture), coupled with stable inter-step generation accuracy, thereby making it capable of being deployed in the real-world patient monitoring use cases.

### Ablation study of effect of AuxC in ClinicalGAN

**Figure 8:**
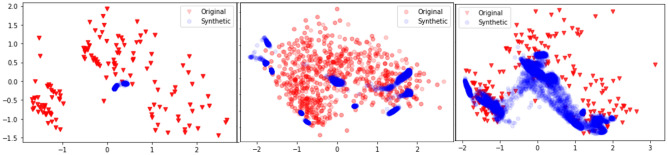
Data variation explained by different generative models with TimeGAN (left), CCGAN (−AuxC) (center) and CGAN(+AuxC) (right). We note mode collapse in TimeGAN and CCGAN (−AuxC) but not in CGAN(+AuxC). This is also reflected in the Alpha-Pr scores: TimeGAN (0.02, CGAN(−AuxC) (0.46) and CGAN(+AuxC) (0.77).

To test the effect of AuxC unit in the ClinicalGAN architecture, we performed an ablation study to note the degree of mode collapse between TimeGAN, CGAN (−AuxC) and CGAN (+AuxC). As summarized in Fig. 8, the tSNE plots of TimeGAN, CGAN (−AuxC) and CGAN (+AuxC) for ADNI test set depict clear mode collapse in TimeGAN and CGAN (−AuxC) experiments. We note that even CGAN (−AuxC) is unable to capture all the variance in patient journeys and focuses only on certain pockets of latent space. Addition of AuxC unit to the ClinicalGAN (CGAN(+AuxC)) architecture led to $~1.5 \times$ of improvement in variance capture (scored 0.77 Alpha-Pr compared to 0.52 of CGAN (−AuxC) as seen in Table 5). The Alpha-Pr scores are a measure of diversity of generated samples, while the tSNE plots visually show the latent space overall between real and generated sequences. The higher Alpha-pr scores along with higher latent space capture seen in tSNE together quantitatively and qualitatively denote that addition of AuxC unit in the architecture aids ClinicalGAN significantly to increase diversity in its generation.

### Drop-off prediction accuracy for active patient monitoring

**Figure 9:**
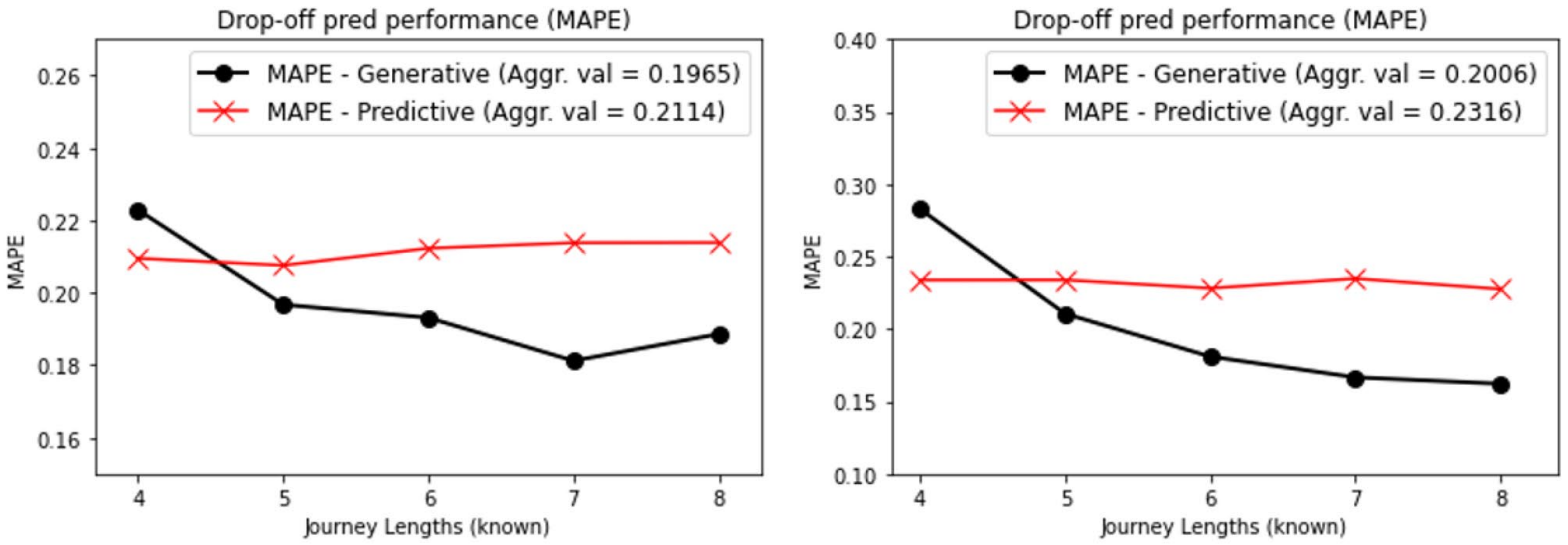
Simulation performance over varying look-ahead steps.

As stated earlier, predictive methods do not capture the inherent stochastic nature of patient journeys, tend to perform poorly in limited data scenarios, and fail to capture the breadth of diversity of journeys. Generative methods tackle all these shortcomings by transferring the learning to a probabilistic setting, which is more suited for the task. To affirm this, we test the performance of ClinicalGAN at the task of drop-off prediction against the predictive method. We set-up this experiment by mimicking the real-world patient monitoring use case where information from the prior steps is available to make drop-off predictions for future steps during test time. For the predictive model, we use XGBRegressor to predict the drop-off visit after each time step. We synthesize its input features by combining the historical information prior to each time step by means of averaging. Meanwhile, for the generative model, we feed the historical information to ClinicalGAN auto-regressively to obtain dynamic drop-off predictions from its Termination network. Since ClinicalGAN is a generative framework, we simulate 1000 journeys for each patient to capture a broad range of probable paths. We then average the drop-off predictions across these simulations to obtain the final predicted drop-off visit for each patient. We report the mean average percentage error (MAPE ; the lower the better) as the evaluation metric for the predictive performance of both methods. In Fig. 9 (left) we depict the performance of both the models on CODR-AD dataset. The y-axis depicts the MAPE performance for each no. of historical visits known at the time of prediction that is reported on the x-axis. We note that ClinicalGAN significantly outperforms the predictive method by 7% MAPE scores aggregated over all the visits (0.199 vs 0.214 MAPE). We also note that ClinicalGAN simulations get better as more historical context is added. This is due to the fact that during the early part of the journey, patients can take any of the multiple probable paths inducing high variance in ClinicalGAN predictions. However, as more historical context gets infused into the system as the trial takes its course, ClinicalGAN is able to adjust its forward-looking simulations and refine its predictions based on the path that the patient has undertaken. We note similar behavior in Fig. 9 (right) that depicts the performance on ADNI dataset. We see that ClinicalGAN again outperforms the predictive method by 13.3% aggregated MAPE scores over all the visits (0.20 vs 0.231 MAPE). Furthermore, we again note that ClinicalGAN’s predictive power gets better as more historical context is added to its input, while the deterministic method is not able to leverage the new information to improve its predictive performance. This attests to the prowess of ClinicalGAN at modeling the patient journeys accurately and delivering reliable results for monitoring patients’ drop-off in clinical trials under a realistic real-world setup.

## Future work

The proposed ClinicalGAN approach has shown to effectively capture temporal data variation and can accurately simulate patient journeys in clinical trials. However, it is currently designed to handle regular interval time-series data only. A natural extension of this work would be to modify the architecture to model irregular time-steps too. Moreover, the current approach shows optimal performance when applied to similar trials within the same therapy areas (TAs). To broaden the applicability, future extension could involve incorporating transfer learning followed by few-shot learning to cross-learn from multiple TAs simultaneously. Furthermore, the proposed ClinicalGAN is evaluated on single modality of structure data (continuous and categorical). However, the architecture can be further expanded to accommodate various other modalities of medical data such as images (X-rays, MRIs, CT-scan and others) and textual data (clinical notes, electronic health records, radiology reports and others) thus providing more comprehensive view of the patient journey. The current framework excels at capturing the multi-variate spatio-temporal relationship between variables within the dataset, allowing it to simulate accurate patient journeys. However, the current approach does not identify the causal relationship between patient drop-offs and the leading events. To address the issue, the current architecture needs to be infused with domain specific knowledge to capture the cause and effect during patient journey and is an area of future research.

## Conclusion

In this work, we proposed ClinicalGAN, a deep generative method capable of generating realistic patient digital twins that exhibit journey characteristics consistent with real patient journeys, while jointly learning the termination conditions based on different journey progressions. We empirically show that ClinicalGAN outperforms the current state-of-the-art AI-based method at the task of multi-variate patient sequence generation across a suite of comprehensive evaluation metrics assessing the generation fidelity, diversity and analytical utility. ClinicalGAN generation quality is also tested over various look-ahead horizons to demonstrate that its stable generation, and retains most of its generative performance over long sequences. Furthermore, the ablation study to isolate the performance gains of different components of ClinicalGAN demonstrates that the carefully designed components contribute to mitigating multiple pitfalls of GAN-based generative models while boosting generation quality significantly. Finally, we demonstrate how ClinicalGAN can be deployed in a real-world clinical trial monitoring setup for predicting patient drop-off. Empirical results over different journey lengths demonstrate that simulation-based drop-off predictions from ClinicalGAN outperform deterministic methods. As part of future work, (a) ClinicalGAN can be extended to inherently handle missing data, (b) leverage more advanced deep learning methods like transformers for sequence modeling and (c) leverage more recent generative methods like diffusion models to better learn the spatio-temporal distributions.

### Supplementary Information

Supplementary Information.

## Data Availability

The Alzheimer’s clinical trial datasets that support the findings of this study are available from CODR-AD and ADNI but restrictions apply to the availability of these data, which were used under license for the current study, and so are not publicly available. The data are not publicly available due to them containing information that could compromise research participant privacy/consent. The CODR-AD data is available on request from the Coalition Against Major Diseases (CAMD) Online Data Repository for AD (CODR-AD)^10,11^. The ADNI data that supports the findings of this study are publicly available in/from ADNI public dataset.
