# Supplementary material for: ClinicalGAN: powering patient monitoring in clinical trials with patient digital twins
Source: Sci Rep. 2024 May 28;14:12236. doi: 10.1038/s41598-024-62567-1 (PMC11133486; doi:10.1038/s41598-024-62567-1)
Supplement: Supplementary file 1 — Supplementary Information. [file 41598_2024_62567_MOESM1_ESM.pdf]

12 Supplementary Information

12.1 Data pre-processing

In this section we will detail the features used in our experiments and pre-processing done for both the datasets. The CODR-AD dataset is received in the standard SDTM format and we develop a streamlined pre-processing pipeline for any SDTM format datasets to standardize this step. Additionally, we used the publicly available version of ADNI dataset from the open-source Kaggle competition. This version of the dataset was already well processed and standardized as part of the public release. We performed some minor processing to align it to the format required by our framework.

12.1.1 CODR-AD

Feature used	SDTM Table Name	Feature Type	Processing in CGAN	Information type
Orientation	QS	0-8 ordinal range	continuous	Longitudinal
Recall		0-3 ordinal range	continuous	Longitudinal
Word recall		0-10 ordinal range	continuous	Longitudinal
Delayed word recall		0-10 ordinal range	continuous	Longitudinal
Alanine aminotransferase	LB	Continuous	continuous	Longitudinal
Alkaline phosphatase		Continuous	continuous	Longitudinal
Aspartate aminotransferase		Continuous	continuous	Longitudinal
Cholesterol		Continuous	continuous	Longitudinal
Creatine kinase		Continuous	continuous	Longitudinal
Creatinine		Continuous	continuous	Longitudinal
Gamma glutamyl transferase		Continuous	continuous	Longitudinal
Hematocrit		Continuous	continuous	Longitudinal
Hemoglobin		Continuous	continuous	Longitudinal
Indirect bilirubin		Continuous	continuous	Longitudinal
Potassium		Continuous	continuous	Longitudinal
Sodium		Continuous	continuous	Longitudinal
ApoE ε allele count		Continuous	continuous	Longitudinal
Triglycerides		Continuous	continuous	Longitudinal
DBP	VS	Continuous	continuous	Longitudinal
SBP		Continuous	continuous	Longitudinal
HR		Continuous	continuous	Longitudinal
Height		Continuous	continuous	Static
Weight		Continuous	continuous	Longitudinal
Age	DM	Continuous	continuous	Static
Geographic region		Categorical	one hot	Static
Race		Categorical	one hot	Static
Sex		Binary	continuous	Static
Initial diagnosis (AD or MCI)		Binary	continuous	Static
Past cardiovascular event	MH	Binary	continuous	Static

Table 6. Variables used in all the experiments on CODR-AD dataset

The CODR-AD database stores data using CDISC standards of Study Data Tabulation Model (SDTM) format which is a widely accepted and used format for storing clinical trial data. The aim of this processing pipeline is to curate any raw SDTM data to a form that can be directly ingested into machine learning algorithms. This implies:

1. Identifying the relevant columns from different tables for analysis.
2. Converting these shortlisted data features to numerical format for all data types: continuous and ordinal features are kept as is, while categorical features are one-hot encoded.
3. Separating the longitudinal data from the static data (patient meta-data) which is captured just once at the start of the trial. They are processed and modeled separately in the framework.
4. Deal with missing values, duplicate rows and other data inconsistencies (eg, having just one visit information, etc).

For CODR-AD, we use a total of 39 static+longitudinal variables in our experiments that are relevant for modeling AD progression. We construct the *static data* for conditional journey generation using the variables present in the **DM** and **MH** tables, and derive 1 feature ourselves. Meanwhile, we construct the *longitudinal data* using the variables present in **QS, VS** and **LB** tables. The details of the feature types of each of the variables used is summarized in Suuplemenrary table 6. The table-level processing is detailed below.

1. DM: We drop the rows that are duplicates of existing ones. Since the "RACE" column has 91% as category "WHITE", and just 0.18% missing values, we impute with mode. We categorize the "ARM" feature into 3 major groups using the mapping below:

- **Placebo Control:** ['PLACEBO', 'PBO', 'Placebo QD', 'BLIND']
- **Active Control:** ['CONTROL', 'ACTIVE ARM 1', 'ACTIVE ARM 2', 'ACTIVE ARM 3', 'ACTIVE ARM 4']
- **Intervention:** ['DRUG 1 DOSE 3', 'DRUG 1', 'DRUG 1 DOSE 2', 'DRUG 1 DOSE 1']

Post this, "ARM" has just 3.5% missing values and "PLACEBO" is by far the most common category value (> 70%), so we impute this column with the mode.

2. MH: We derive feature called "past cardiac event" from this table by using the "MHTERM" variable in the original table. The derived feature is a binary feature indicating if the respective patient has had any kind of cardiac event in the past.
Finally, we merge the DM and MH tables on patientID to get 8220 common patients as part of the final **static** dataset.

3,4,5. QS, VS, LB: We follow the same pre-processing steps for features from these tables. We extract the longitudinal information of the variables mentioned in table 6 for all the patients. We impute missing values by the mean of each patient for the respective features. Any rows still containing missing values after this operation are dropped from the final processed versions.
Finally, we merge the QS, VS and LB tables on patientID and visit number to get 1361 common patients who have complete longitudinal data as part of the final **sequential** dataset. Note that we prune the 8220 patients in static dataset to the 1361 patients found in sequential data to get same number of patients across the two datasets.

12.1.2 ADNI

Feature used	Feature Type	Processing in CGAN	Information Type
AGE	Continuous	continuous	Static
Gender	Categorical	one hot	Static
Education Level	Categorical	one hot	Static
Diagnosis	Categorical	one hot	Static
ApoE ε allele count	Continuous	continuous	Longitudinal
Clinical Dementia Rating	Ordinal	continuous	Longitudinal
Mini-Mental State Examination	Ordinal	continuous	Longitudinal
Alzheimer's Disease Assessment Scale-Cognitive Subscale	Ordinal	continuous	Longitudinal
Ventricle volume	Continuous	continuous	Longitudinal
Hippocampus volume	Continuous	continuous	Longitudinal
Whole brain volume	Continuous	continuous	Longitudinal
Entorhinal volume	Continuous	continuous	Longitudinal
Fusiform volume	Continuous	continuous	Longitudinal
Midtemp volume	Continuous	continuous	Longitudinal

Table 7. Variables used in all the experiments on ADNI dataset

The Alzheimer's Disease Neuroimaging Initiative (ADNI) data[9] consists of of clinical trial longitudinal data from from four studies. These trials have been enrolling subjects across the AD disease spectrum since 2004, focusing primarily on MCI and cognitively normal subjects. We use a publicly available version of the dataset sourced from the 2022 Kaggle competition - ADNI public dataset. The details of the feature types of each of the variables used is summarized in Table 7.

The open dataset is clean and does not contain any missing values. We one-hot encode the categorical features and segregate the data into **static** and **sequential** datasets. The final dataset contains 1226 patients' longitudinal journeys.

Dataset	Model name	ACD ↓	FID ↓	Alpha-PR ↑	Discriminator Score ↓			Next-step Prediction ↓	
					AUCROC	PRAUC	F1	Real MSE	Gen MSE
CODR-AD	TimeGAN[5]	0.557	7.03	0.02	0.881	0.913	0.821	0.013	0.099
	CGAN (+AuxC) - LSTM	<u>0.249</u>	**0.85**	<u>0.71</u>	<u>0.534</u>	<u>0.540</u>	<u>0.692</u>	0.013	**0.021**
	CGAN (+AuxC) - GRU	**0.246**	<u>0.87</u>	**0.72**	**0.515**	**0.535**	**0.674**	0.013	<u>0.023</u>
ADNI	TimeGAN[5]	0.558	7.37	0.02	0.99	0.99	0.99	0.015	0.16
	CGAN (+AuxC) - LSTM	<u>0.211</u>	<u>1.14</u>	<u>0.77</u>	**0.76**	<u>0.69</u>	<u>0.77</u>	0.015	**0.025**
	CGAN (+AuxC) - GRU	**0.202**	**1.07**	**0.77**	**0.77**	**0.68**	<u>0.77</u>	0.015	<u>0.027</u>

Table 8. Generation quality metrics results on TimeGAN and CGAN(+AuxC) for both LSTM and GRU best models for CODR-AD and ADNI datasets over 1000 simulations for each patient in the test set. ACD represents the Avg_corr_diff scores. Values in **Bold** represent best scores and in <u>Underline</u> represent second best scores within a dataset. ↑ denote higher scores are better for that metric, and ↓ the vice versa.

12.2 Effect of latent dimension on AE

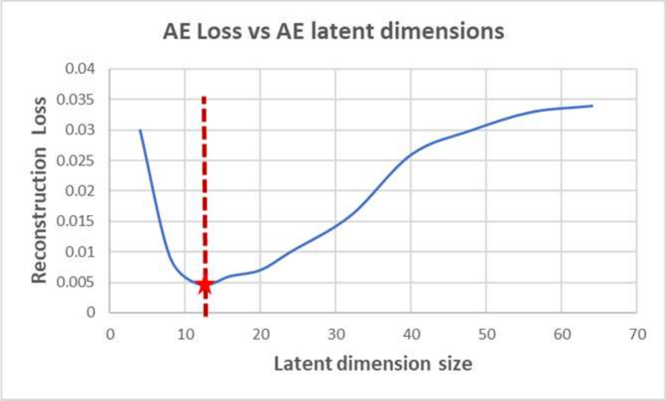

Figure 10. Test set AE loss (reconstruction loss) for different AE latent dimensions from 4 to 64 with resolution of 4 units.

AE struggles to encode information effectively if the latent dimension is too low. For higher latent dimension size, the AE overfits on training samples and performs poorly on unseen test set. As per the experimentation results in Fig 10 above, we have used latent dimension of 12 for CODR-AD dataset.

12.3 Choice of GRU vs LSTM

During our experiments, we noted minor difference in performance of ClinicalGAN with either a GRU or LSTM (demonstrated in supplementary figure 8) as the temporal unit. This denotes that the ClinicalGAN architecture is not sensitive to the choice of the RNN used and tends to perform similarly in both. We chose GRU in the final model as it tends to perform slightly better overall, while also having lower computation footprint.

12.4 ClinicalGAN pseudo-code

Algorithms 1 below details the pre-trainig phase while Algorithm 2 details the joint training phase of ClinicalGAN. Note that the individual losses mentioned in the procedures below are defined in detail in Section 3.3. To summarize, ClinicalGAN first independently pre-trains the AE, Tr and S networks before jointly training them with G,D and AuxC in the final procedure.

12.5 Additional Results

We provide additional results from Section 5.3 below. Specifically, Supplementary figure **??** and Supplementary figure 12 demonstrate the univariate KDEs of TimeGAN and ClinicalGAN on ADNI dataset respectively. Similarly, Figure 13 denotes the correlation plots of TimeGAN and ClinicalGAN on the ADNI dataset.

Algorithm 1 ClinicalGAN training

x_{st} is the static data matrix
x_{seq} is the sequential data matrix
lr is the learning rate
Initialize AE, Tr, S, G, D and AuxC network components
procedure AE PRETRAIN(x_{st}, x_{seq}, epochs, lr)
    while AE pretrain epochs **do**
        AE optimized minimizing the Reconstruction loss
    end while
end procedure
procedure SUPERVISOR PRETRAIN(x_{st}, x_{seq}, epochs, lr)
    while Supervisor pretrain epochs **do**
        S optimized minimizing the MSE loss
    end while
end procedure
procedure TERMINATION NETWORK PRETRAIN(x_{st}, x_{seq}, epochs, lr)
    while TerminNetw pretrain epochs **do**
        Tr optimized minimizing cross-entropy loss
    end while
end procedure
procedure JOINT TRAINING(x_{st}, x_{seq}, epochs, lr)
    while Joint pretrain epochs **do**
        All network components optimized together using the joint training loss end-to-end
    end while
end procedure

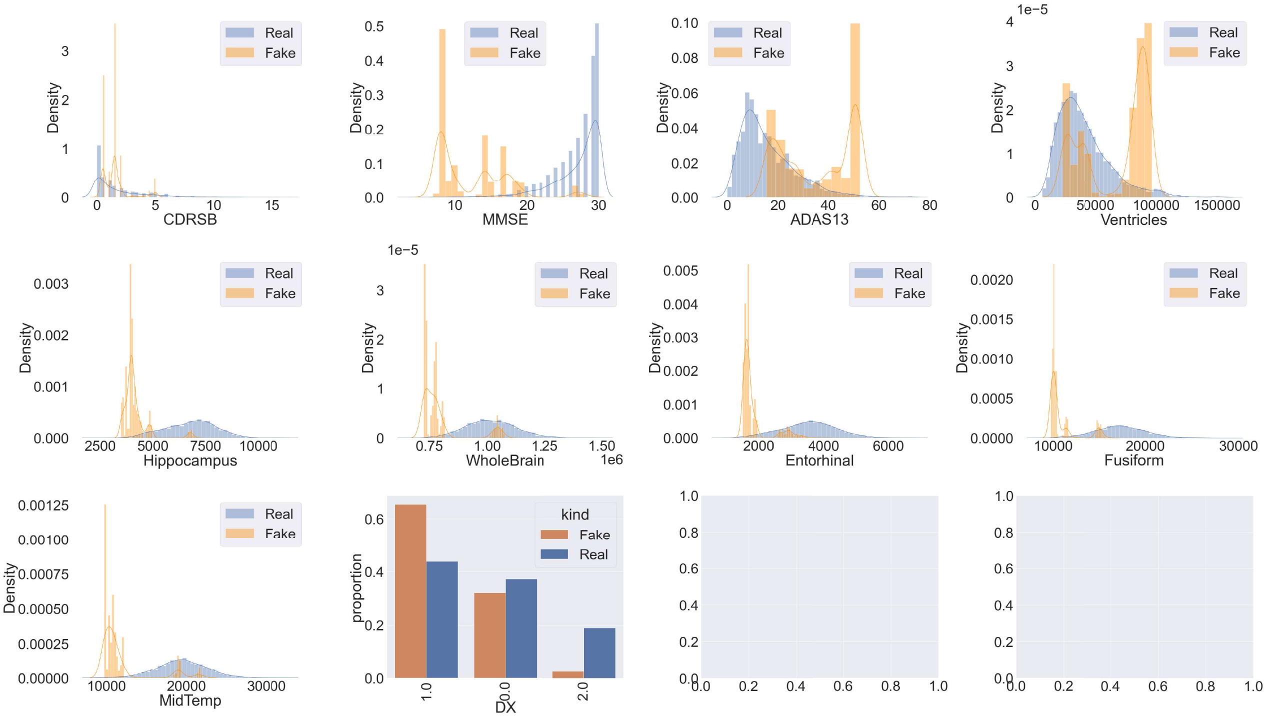

Figure 11. KDEs of TimeGANon ADNI dataset.

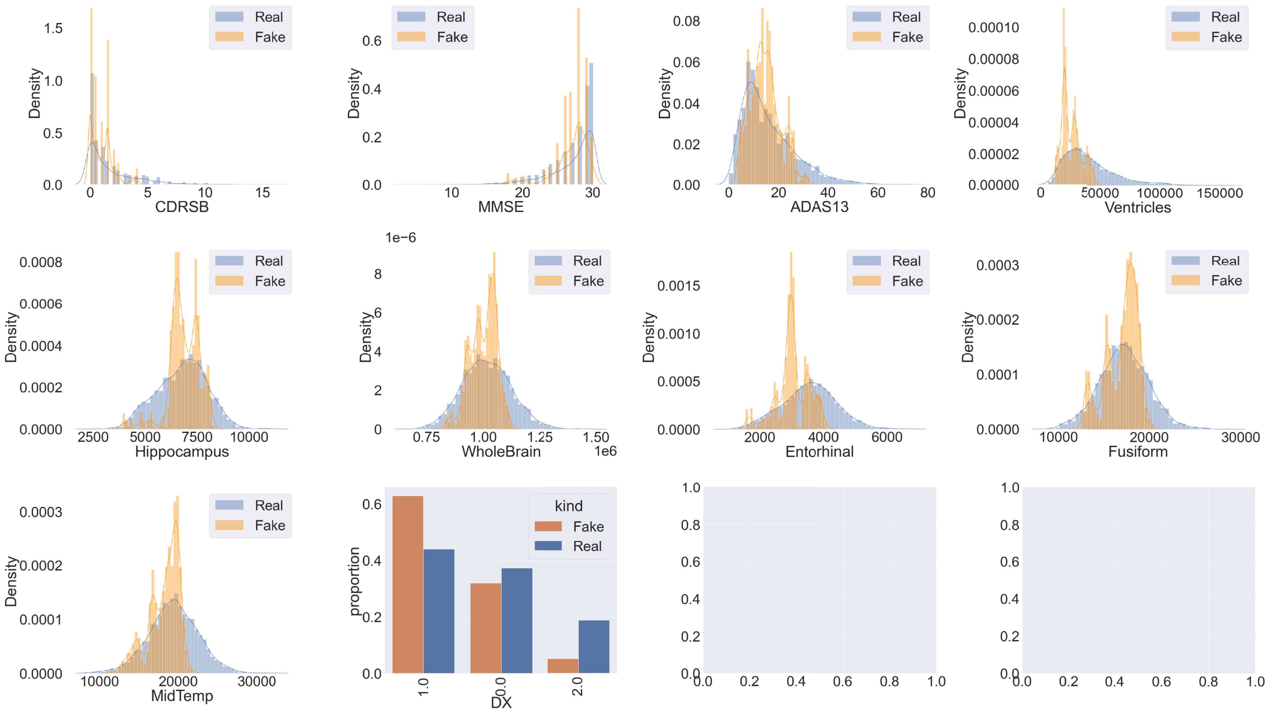

Figure 12. KDEs of ClinicalGAN on ADNI dataset.

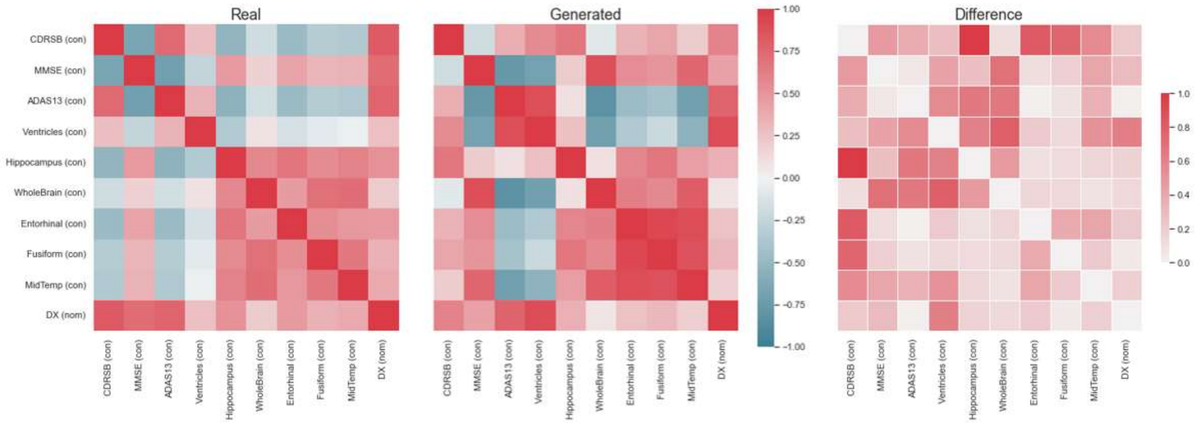

(a) TimeGAN

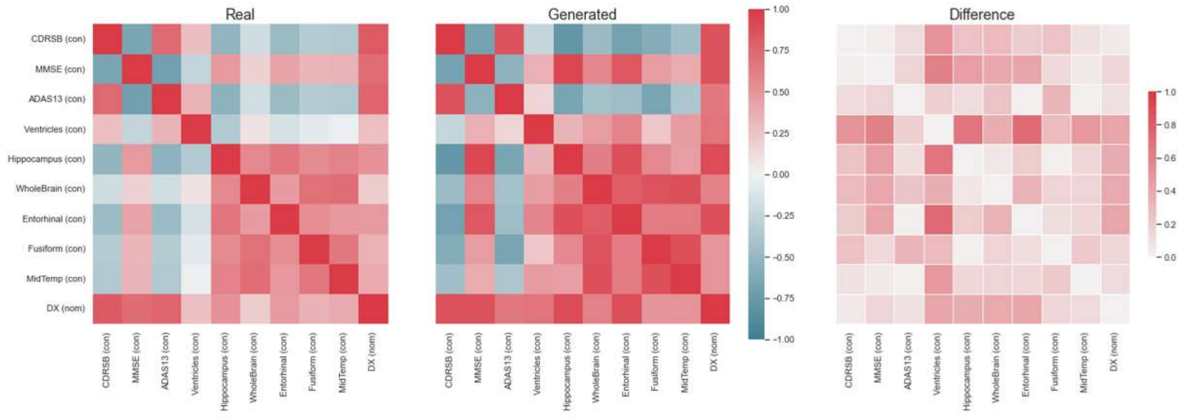

(b) ClinicalGAN

Figure 13. Correlation heatmaps of ClinicalGAN and TimeGAN models on ADNI dataset.
